# Behavior of Occupied and Void Space in Molecular Crystal Structures at High Pressure

**DOI:** 10.1021/acs.cgd.1c01427

**Published:** 2022-03-22

**Authors:** Cameron J. G. Wilson, Tomas Cervenka, Peter A. Wood, Simon Parsons

**Affiliations:** † Centre for Science at Extreme Conditions, School of Chemistry, The University of Edinburgh, King’s Buildings, West Mains Road, Edinburgh EH9 3FJ, U.K..; ‡ The Cambridge Crystallographic Data Centre, 12 Union Road, Cambridge CB2 1EZ, U.K.

## Abstract

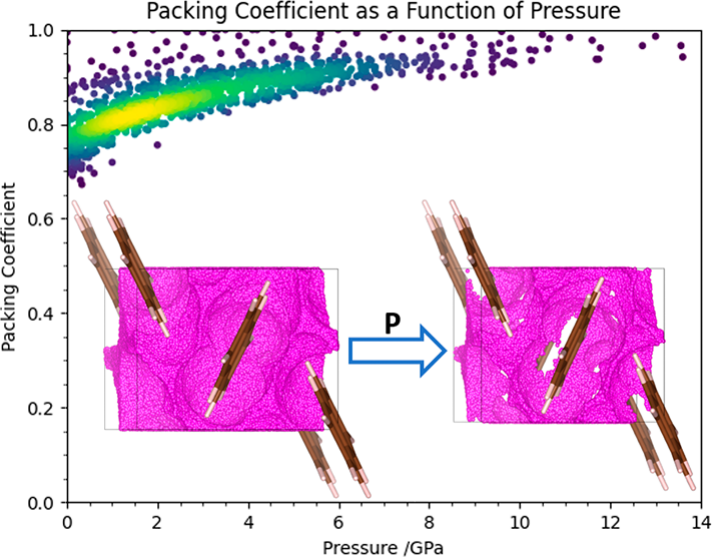

We report a Monte Carlo algorithm for calculation of occupied (“network”) and unoccupied (“void”) space in crystal structures. The variation of the volumes of the voids and the network of intermolecular contacts with pressure sensitively reveals discontinuities associated with first- and second-order phase transitions, providing insights into the effect of compression (and, in principle, other external stimuli) at a level between those observed in individual contact distances and the overall unit cell dimensions. The method is shown to be especially useful for the correlation of high-pressure crystallographic and spectroscopic data, illustrated for naphthalene, where a phase transition previously detected by vibrational spectroscopy, and debated in the literature for over 80 years, has been revealed unambiguously in crystallographic data for the first time. Premonitory behavior before a phase transition and crystal collapse at the end of a compression series has also been detected. The network and void volumes for 129 high-pressure studies taken from the Cambridge Structural Database (CSD) were fitted to equation of state to show that networks typically have bulk moduli between 40 and 150 GPa, while those of voids fall into a much smaller range, 2–5 GPa. These figures are shown to reproduce the narrow range of overall bulk moduli of molecular solids (*ca.* 5–20 GPa). The program, called CellVol, has been written in Python using the CSD Python API and can be run through the command line or through the Cambridge Crystallographic Data Centre’s Mercury interface.

## Abstract 2

The overall volume of a crystal structure can be partitioned into occupied (“network”) and unoccupied (“void”) space. Trends with pressure are very sensitive indicators of phase transitions, correlating closely with spectroscopic data, and can even reveal effects premonitory to loss of long-range order.

## 1 Introduction

**Figure 1:**
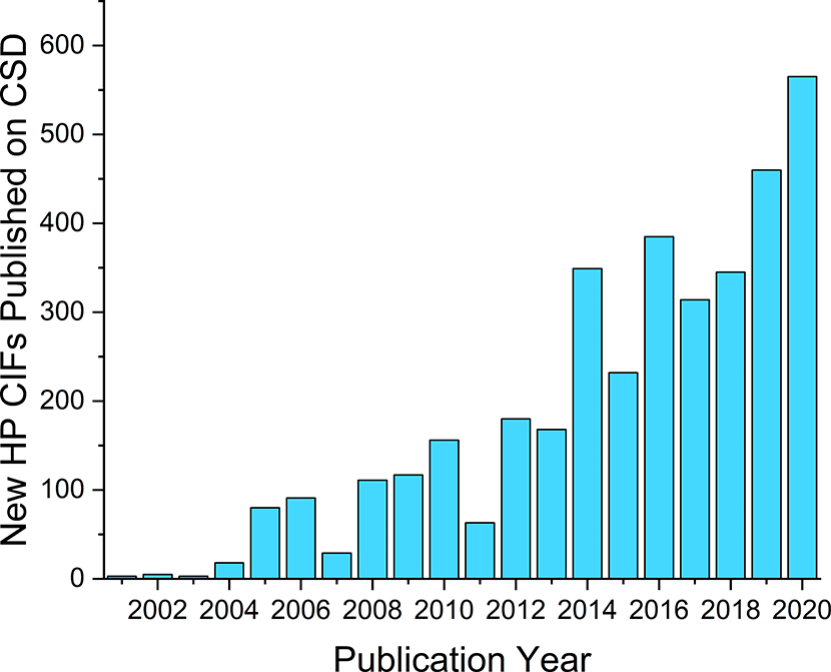
Number of annual depositions of high-pressure crystal structures in the CSD (version 5.42 with updates up to and including February 2021).

Use of high pressure to investigate the polymorphism and the mechanical properties of different classes of intermolecular interaction is increasingly popular, Figure 1 showing the number of depositions on the Cambridge Structural Database (CSD) of structures determined at above 0.1 GPa.^1^ An observed crystal structure represents a minimum in free energy (*G* = *U* + *PV* – *TS*, where *G* is the free energy, *U* is the internal energy, *P* is the pressure, *V* is the volume, *T* is the temperature, and *S* is the entropy). Under ambient conditions, this corresponds to optimization of the balance between internal energy (determined by formation of favorable intramolecular geometry and intermolecular contacts such as H bonds) and entropy, but at high pressure, the need to minimize volume becomes increasingly important. For a system of constant composition, the volume always decreases with increasing pressure and first-order phase transitions occurring at high pressure are always accompanied with a decrease in volume. Volume minimization is the dominant driving force in almost all high-pressure phase transitions, although the relief of unfavorably compressed contacts can also play a role.^2^ For example, over the course of the phase transition between l-serine-I and l-serine-II that occurs above 4.8 GPa, a decrease in volume overcomes a destabilizing lattice energy change.^3^ Analysis of volume changes is therefore critical in the interpretation of phase transitions at high pressure.

The volume of a crystal structure is made up of contributions from the atoms, ions or molecules, their network of interactions, and the interstitial voids. In close-packed hard-sphere structures, the fraction of the volume occupied by atoms is 0.72 (the packing coefficient), with unoccupied interstitial voids comprising the remainder.^4^ Kitaigorodsky recognized that the underlying topology of molecular crystal structures is also often found to correspond to close packing or body-centered cubic arrangements,^5^ a conclusion which has been extensively explored by Peresypkina and Blatov using formal topological analysis based on Voronoi–Dirichlet partitioning.^6^ Accordingly, the packing coefficients of molecular structures are broadly similar to those of hard-sphere structures and usually fall into the range 0.6–0.8 at ambient pressure.^4^

Some crystal structures, such as those of metal organic frameworks, contain large void spaces capable of accommodating guest species, including gases, common solvents, and even relatively large molecules such Keggin anions in porous chromium terephthalate MIL-101 (OCUNAC, unit cell volume = 701 860 Å^3^) with pore volumes up to ∼20 600 Å^3^.^7^ At least 20% of the total unit cell volume is unoccupied at ambient pressure even in non-porous molecular crystals. This space is distributed over small interstitial sites around and between the molecules and is not accessible to guest species.

There are a number of algorithms available to evaluate occupied and void space in both classes of molecular crystal structures. Some of these use a spherical probe, which is rolled over the van der Waals surface of a molecule, mapping where the surfaces of the probe and molecule meet. An alternative “accessible surface” can be defined by mapping the position of the center of the probe.^8^ A topological approach has been described by Blatov^9^ where voids are constructed by first partitioning the crystal structure into Voronoi–Dirichlet polyhedra (VDPs) using the atomic positions and then reapplying the same partitioning algorithm to both the atoms and the vertices of the original VDPs. The new VDPs centered on the original vertices then define the voids. Gavezzotti introduced a method where the unit cell volume is divided into pixels of typical volume of 0.001 Å^3^; the void volume is the fraction of pixels not within the van der Waals radius of an atom multiplied by the total unit cell volume.^4^ Spackman and co-workers have partitioned structures into occupied and void regions using a value (typically 0.002 au) of the promolecule electron density to define the molecular surfaces.^10^ The methods of Blatov, Gavezzotti, and Spackman sample the volume within the nooks and crannies on a molecular surface that might be missed by the rolling probe method. The methods described have been implemented in numerous crystallographic and molecular graphics programs including Mercury, PLATON, OLEX-2, X-seed, CrystalExplorer, ATOMS, Oscail, and TOPOS.^8,10,11^

Molecular structures often respond to high pressure initially by compression of void space, as shown by high-pressure analysis of the voids in α-d-mannose using the rolling probe method.^12^ The inference is that the void space is softer than the network of inter and intramolecular contacts, and one of the aims of this paper is to quantify this difference. We will describe a simple algorithm for partitioning the volume of a unit cell (*V*) into contributions from the occupied network of inter- and intra-molecular interactions, *V*_net_, and unoccupied void volume, *V*_void_, which has the advantage that it is easily combined with tools available in the CSD Python API.^1^ The variations in *V*_net_ and *V*_void_ with pressure show how the intermolecular interactions and the void space combine to determine the overall compressibility of a material. We will also show how discontinuities in this behavior can be used to detect subtle structural phase transitions and even premonitory behavior.

## 2 Computational Procedures

### 2.1 Network and Void Volume Calculations

The aim of the computational procedure described below was to partition the volume of a unit cell (*V*) into regions occupied by atoms and their network of intermolecular contacts (*V*_net_) and regions that consist of unoccupied void space (*V*_void_). The values of the network and void volumes were evaluated in a Monte Carlo procedure, which can be illustrated by the calculation of the volume of a sphere of radius *r.* A cube of known volume, *V*_cube_, which encloses the sphere, is populated at random with a large number of *n* points. A point will lie within the sphere if its distance from the center of the sphere is less than *r*. Hence, if there are *n*_s_ points within the sphere, the quantity (*n*_s_/*n*)*V*_cube_ converges to the volume of the sphere as *n* increases.

The same procedure was used to evaluate the void and network volumes in a crystal structure. Points at fractional coordinates [*x*, *y*, *z*] were generated from a uniform probability distribution between 0 and 1. The number of points generated, *n*, was of order 10^6^. The distance from each point to atoms within and just beyond (see below) the edges of the unit cell was calculated. Points which were beyond the van der Waals radii of the atoms were defined as belonging to the voids, while those within the radius of any atom belong to the network of molecules and contacts. The van der Waals radii used were those given by Alvarez.^13^ If the number of points in the network volume is *n*_net_, then
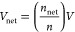
1
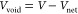
2

The input to the procedure for the calculation is a crystallographic information file (CIF). The first step of the calculation is to normalize X···H distances to neutron values if the structure had been determined using X-rays. A list of atoms is generated consisting of the contents of the reference unit cell and any atoms within the van der Waals radius of the largest atom (*R*_max_) of the unit cell edges. Inclusion of these atoms is necessary because a point inside the unit cell may sit within the van der Waals radius of an atom just outside. An atom was included if |*x*| < *R*_max_/*a*, |*y*| < *R*_max_/*b*, and |*z*| < *R*_max_/*c*, where *a*, *b*, and *c* are unit cell dimensions. The calculation then proceeds, as described above.

The precision of the volume estimations obtained from the Monte Carlo procedure varies as √*n*,^14^ and because the calculation is based on random numbers, the values of the void and network volumes differ between individual runs. The standard deviation, σ(*V*_net_), of the network volume can be estimated by performing multiple runs. If after three such runs, the value of σ(*V*_net_)/*V*_net_ is less than a target value, the calculation finishes, otherwise the calculation will continue to a user-defined maximum. The precision is defined by the “population” standard deviation of the network volumes, rather than the standard deviation of the mean network volume. It does not decrease with the number of repeated runs but does become better defined. If the required precision has not been achieved, a warning is printed, and the calculation should be repeated with a larger value of *n*. The target value of σ(*V*_net_)/*V*_net_ used for this work was 0.1% and was achieved with a million points in three runs for almost all structures (see Figure S1 in Supporting Information).

The total precision of the volume estimates has two sources. One is that described above, arising from the reproducibility of the Monte Carlo calculations. The other arises from the precision of the structural parameters themselves. These can be propagated into the final volume estimates in a second Monte Carlo procedure in which multiple structural models are generated by perturbing each atomic coordinate with independent Gaussian random deviates taken from a distribution with a mean of zero and a standard deviation equal to the coordinate standard uncertainty.^15^ The network and void volume can be calculated for each perturbed structure, generating a distribution. The mean and standard deviation of this distribution, which reflects the scatter obtained from both Monte Carlo procedures, are then taken as the network volume and its standard deviation.

In this work, the standard deviations of the volumes quoted for the structures in Section 3.3 were generated using 100 perturbed sets of coordinates at each pressure point. The structures analyzed in Sections 3.1 and 3.2 are the results of density functional theory (DFT) optimizations, for which no coordinate standard uncertainties are available, and in these cases, the precision is based solely on the spread of the volume estimates from a single set of coordinates. When the number of points used in the Monte Carlo volume calculations is chosen to give σ(*V*_net_)/*V*_net_ = 0.1%, the spread of values obtained with coordinate error propagation is not very different from that obtained from a single structure, suggesting that the spread is dominated by the reproducibility within the individual Monte Carlo volume calculations. If the number of points is chosen to reduce σ(*V*_net_)/*V*_net_ significantly below 0.1%, or if low precision structures are analyzed, then the propagated error would be expected to become more significant.

Our approach for volume calculations is similar to the algorithm outlined by Gavezzotti for calculation of molecular volumes. In Gavezzotti’s approach, the unit cell volume would have been divided into pixels with a volume in the region of 0.001 Å^3^ and each one classified according to whether it was or was not within the van der Waals radius of an atom. This calculation is more efficient than our Monte Carlo method for small unit cells, but becomes slower for large unit cells. For example, a unit cell with a volume of 5000 Å^3^ would require calculations for 5 million pixels, as opposed to multiple Monte Carlo runs of 1 million pixels, which are often found to complete in three runs. The Monte Carlo method also enables iteration, enabling a target precision to be defined. The use of dimensionless points instead of pixels (or a rolling probe) may also sample small features on molecular surfaces.

The algorithm has been implemented in a program called CellVol, written in Python using the NumPy library and functions available in the CSD Python API. Investigations were made into speeding up the calculation based on their asymmetric unit rather than the unit cell. The CSD Python API does not currently include a function for identifying the coordinate limits of the asymmetric unit of a space group, but testing the algorithm using the CrysFML Fortran library,^16^ which does have this feature, did not lead to a sufficient increase in speed or precision to make consideration of space group symmetry worthwhile. The majority of structures studied at high pressure have unit cell volumes of less than 1000 Å^3^, and the calculation time shows no appreciable dependence of volume of the unit cell in this range. At higher volumes, there is a roughly linear dependence. Calculations were shown to complete within 5 min for 97% of structures on the CSD using a modest desktop personal computer with an Intel Core i7-9700 CPU with a base speed of 3.00 GHz (see Supporting Information, Figure S2).

The program can be invoked from within Mercury or the command line for both CSD entries and user-supplied CIFs and may be used for single or multiple structures. The values of *n*, the maximum number of runs, and the required precision can be set by the user *via* a GUI or as command line arguments. The code is available and maintained in the open-source format from https://github.com/CwilsonEd/CellVol.

### 2.2 Calculation of the Network and Void Bulk Moduli for Compression Studies in the CSD

The isothermal bulk modulus of a compound is the inverse of its compressibility and is defined as
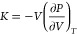
3where *K* = bulk modulus, *V* = volume, *P* = pressure, and *T* = temperature.

Network and void bulk moduli were calculated using the EoSFit program by equation of state (EoS) fitting of the variation of void or network volumes with pressure^17^ for compounds in the CSD for which variable pressure data are available, the list being taken from the study by Giordano *et al.* 2019.^18^ Ambient pressure reference structures were added to the high-pressure refcode sets where necessary. Structures, which contained no coordinates, had unhandled errors, or contained disorder, were removed. Refcode sets with less than five members were also removed. The final data set contained 1472 separate refcodes in 129 refcode families. Systematic differences were often seen when several separate studies were combined, and where possible data were taken from contiguous sets of measurements from a single investigation. Network and void volumes were scaled to *Z* (the number of formula units per unit cell) to allow a comparison across phase transitions where *Z* changes. The results of these calculations are given in Table S1 in the Supporting Information.

### 2.3 Periodic DFT Calculations

High-pressure crystal structures of naphthalene were optimized using periodic DFT using the CASTEP program^19^ in order to remove geometric variation related to instabilities in Rietveld refinements. The published structures were used for starting coordinates with the unit cell parameters and space group held fixed to those that were determined experimentally. A basis set cutoff of 950 eV was used with a Perdew–Burke–Ernzerhof (PBE) exchange–correlation functional with “on the fly” pseudopotentials embedded in the program. The **k**-point spacing was 0.08 Å^–1^. These parameters converged the total energy to less than 0.1 meV per atom. For the geometry optimization, a tolerance of 5 × 10^–6^ eV atom^–1^ was used for energy convergence with a maximum force tolerance of 0.01 eV Å^–1^ and a maximum displacement tolerance of 5 × 10^–4^ Å.

### 2.4 Pixel Calculations

Intermolecular interaction energies in naphthalene were calculated with the Pixel method^4,20^ using the MrPixel interface.^21^ Gaussian-09^22^ was used to calculate the electron density at the MP2 level of theory with the 6-31G** basis set. The molecular electron density was calculated on a grid of 0.08 × 0.08 × 0.08 Å^3^, and a condensation level equal to 4 was used for the Pixel calculations out to a cluster radius of 14 Å. The total energy of each of the contacts was taken as the sum of the Coulombic, polarization, dispersion, and repulsion interactions.

## 3 Results

### 3.1 Detection of First-Order Transitions in l-Histidine

**Scheme 1:**
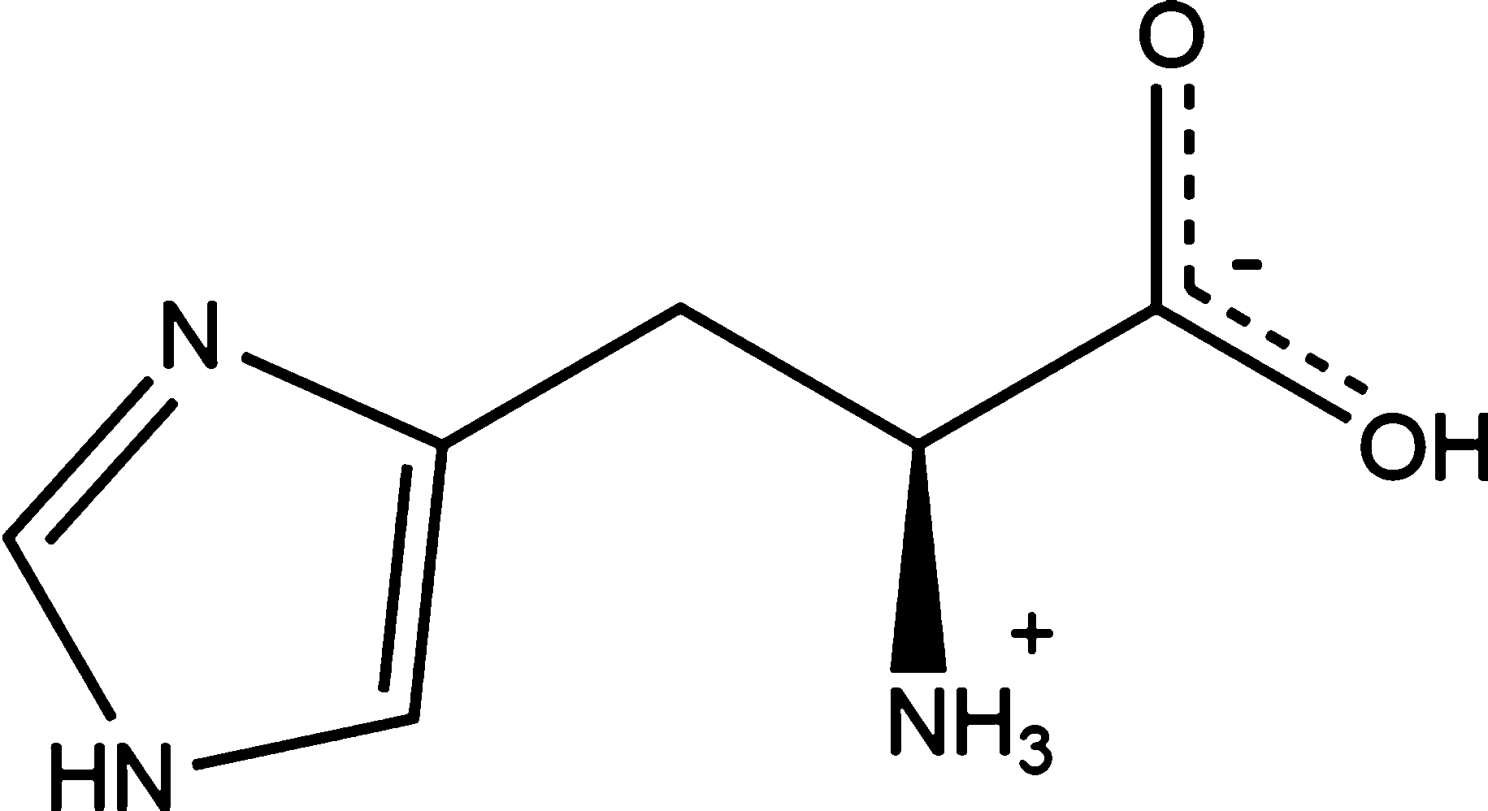
Molecular Structure of l-Histidine

The amino acid l-histidine (Scheme 1) has two stable ambient pressure polymorphs, an orthorhombic (*P*2_1_2_1_2_1_) and a monoclinic (*P*2_1_) form, both of which were recently studied to between 6 and 7 GPa.^23^ High-pressure phase transitions were identified for both, at 4.5 GPa for the orthorhombic form and at 3.1 GPa for the monoclinic form. The space group symmetry was preserved in both transitions, which were driven by a reduction in volume. Despite being shown to have remarkably similar ambient pressure interaction energies, crystal packing, and molecular conformations, the two polymorphs were shown to be different in their response to compression. Prior to the phase transition in the monoclinic form, chains formed by NH···O interactions shear relative to one another, but the same compression mechanism cannot occur in the orthorhombic form without a change in space group symmetry.

**Figure 2:**
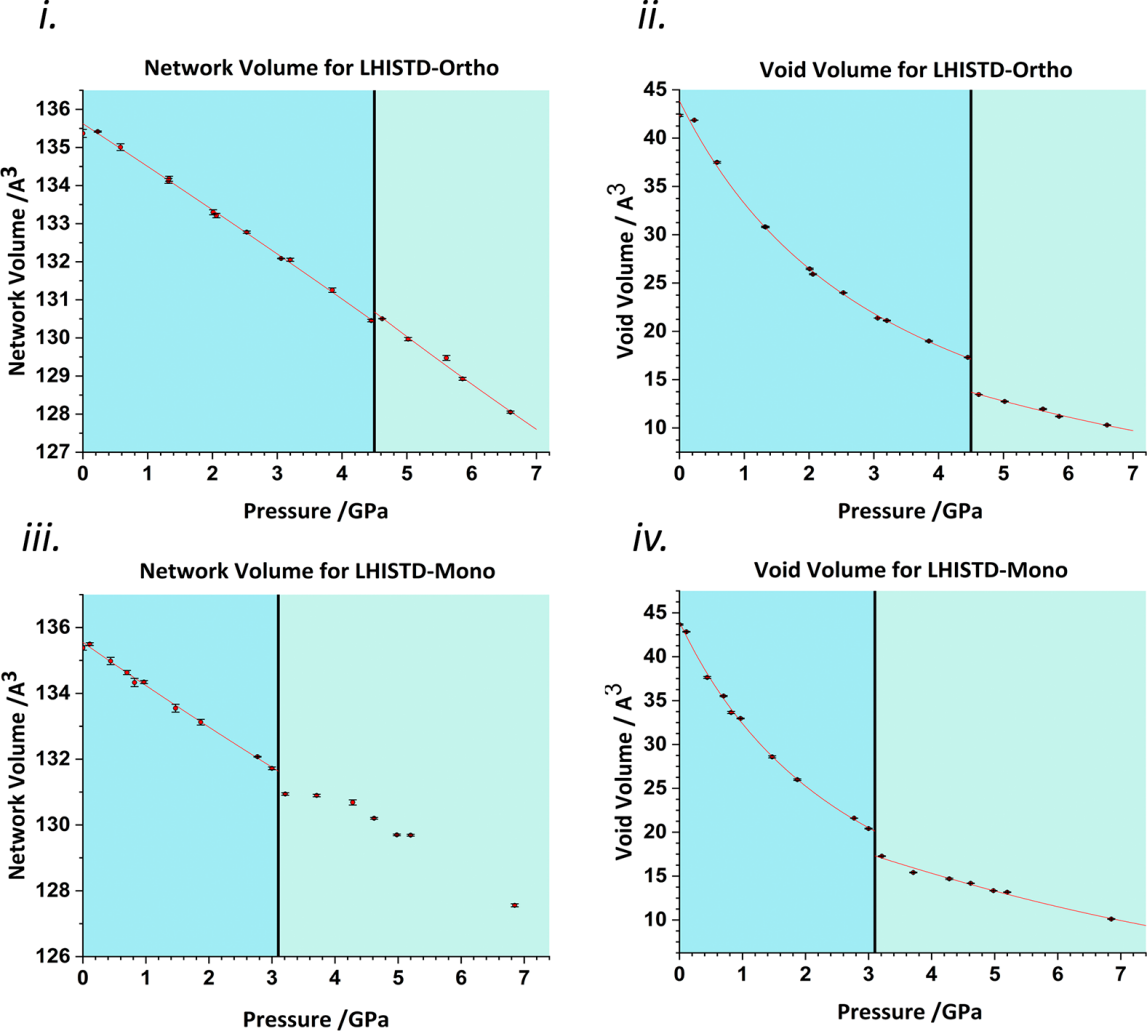
Void analysis for l-histidine (i). Form I network volume against pressure, a third-order Birch–Murnaghan EoS is fitted before the transition and a second-order Birch–Murnaghan EoS afterwards. (ii) Form I void volume against pressure, third-order Vinet EoSs are fitted before and after the transition. (iii) Form II network volume against pressure, a third-order Birch–Murnaghan EoS is fitted before the transition. (iv) Form II void volume against pressure, third-order Vinet EoSs are fitted before and after the transition. Black vertical lines mark phase transitions.

Volume and packing energy analyses in the original study were based on experimentally determined coordinates, which had been optimized by periodic DFT. The same coordinates were used in the present work, with the results shown in Figure 2(i–iv). For the orthorhombic form, the network shows a small increase of 0.05 Å^3^ per molecule at the phase transition at 4.5 GPa (Figure 2(i)), which is compensated by the reduction of the void volume of 3.84 Å^3^ per molecule (Figure 2(ii)). This increase in the network volume signals a rearrangement at the transition, which enables the molecules to make more efficient use of the void space. Fitting of the network volume using second- and third-order Birch–Murnaghan EoSs before and after the phase transition reveals a reduction in bulk modulus following the transition from 121(5) to 83(5) GPa. The 22.2% change in void volume at the transition compares to a unit cell reduction of only 2.6%, showing that discontinuities can be considerably larger in the component (*i.e.*, network or void) volumes than that in the total volume.

For the monoclinic form, the change is more substantial. At the phase transition (3.1 GPa), the network volume undergoes a discontinuous drop (Figure 2(iii)). This reduction in the network accounts for 0.78 Å^3^ per molecule, accompanied by a 3.14 Å^3^ per molecule reduction in the void space. The reduction of volume in the void (Figure 2(iv)) appears to be the driving force for this transition with a reduction of 15.4% of the void on transition compared to the 2.6% reduction in the total unit cell volume. A Vinet EoS fit for the void volume before and after the transition reveals an increase in void bulk modulus from 2.9(2) to 9(3) GPa. The change in the network bulk modulus is harder to discern because the trend, as shown in Figure 2(iii), after the transition does not follow the typical functional form of a Birch–Murnaghan or Vinet EoS, but we estimate that it increases marginally from 101(9) to 139 GPa using a third-order Birch–Murnaghan EoS before the transition and eq 4 afterwards.^23^
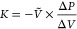
4

Despite the similarity of the geometries and energies of the intermolecular interactions in the two polymorphs, the way in which the networks and voids interact to minimize the volume is different. These results correct the analysis presented by Novelli *et al.* 2020,^23^ where both transitions were described as occurring with an increase in network volume and premonitory behavior detected in the monoclinic form. The trends reported previously were the result of a programming error.

### 3.2 Detection of Subtle High-Pressure Second-Order Transitions

Compression studies of molecular materials using Raman and other forms of spectroscopy can reveal structural rearrangements, which are difficult to detect in crystallographic data. The existence of a phase transition between 2 and 4 GPa in naphthalene has been the subject of debate in the literature for over 80 years, owing to the importance of polycyclic aromatic hydrocarbons in oil, tar, and coal deposits in the Earth’s crust and the relevance of their mechanical behavior in modeling the geophysics of planets and their moons in the Solar System.^24^ An “unmistakable” but “sluggish” transition was first identified by Bridgman in 1938 using volumetric measurements to 3.0 GPa.^25^ A study by Block *et al.* 1970 detected this transition in the range 2–3.5 GPa through optical observation of a sample in a diamond anvil cell.^26^ However, this was followed by a volumetric study by Vaidya and Kennedy in 1971 and a Raman study by Nicol *et al.* in 1975 which both found no evidence of a structural phase transition up to 4 and 3.6 GPa, respectively.^27,28^ An infrared study by Hamann *et al.* 1978 provided support for the hypothesis of a sluggish transition by demonstrating a discontinuous shift in all bands in the range 2–4 GPa.^29^ A Raman study over the same pressure range by Meletov *et al.* 2013 proposed that discontinuous changes in relative phonon frequencies signaled a phase transition near 3.5 GPa.^30^ Most recently, a study by O’Bannon and Williams using both Raman and infrared spectroscopy confirmed a subtle transition at 2–3 GPa,^24^ the greater sensitivity of the infrared data when compared to the Raman data in this paper being suggested as an explanation for the lack of transition observed in Nicol’s study. The same study identifies a further phase transition above 15 GPa, which involves dimerization or polymerization of the molecules.

The CSD contains two crystallographic studies on naphthalene.^31,32^ The first of these studies (NAPHTA19-22) was a single-crystal X-ray study by Fabbiani *et al.* 2006, which features four high-pressure points to 2.1 GPa;^31^ no transitions were observed, as would be expected from the spectroscopic data. The second was a synchrotron powder study (NAPHTA39-48) by Likhacheva *et al.* in 2014, which detected anisotropic compression but no transitions up to 5.60 GPa.^32^ These data were also included in a later study by the same authors, extending the pressure limit to 13 GPa with all data fitted to a single EoS.^33^

**Figure 3:**
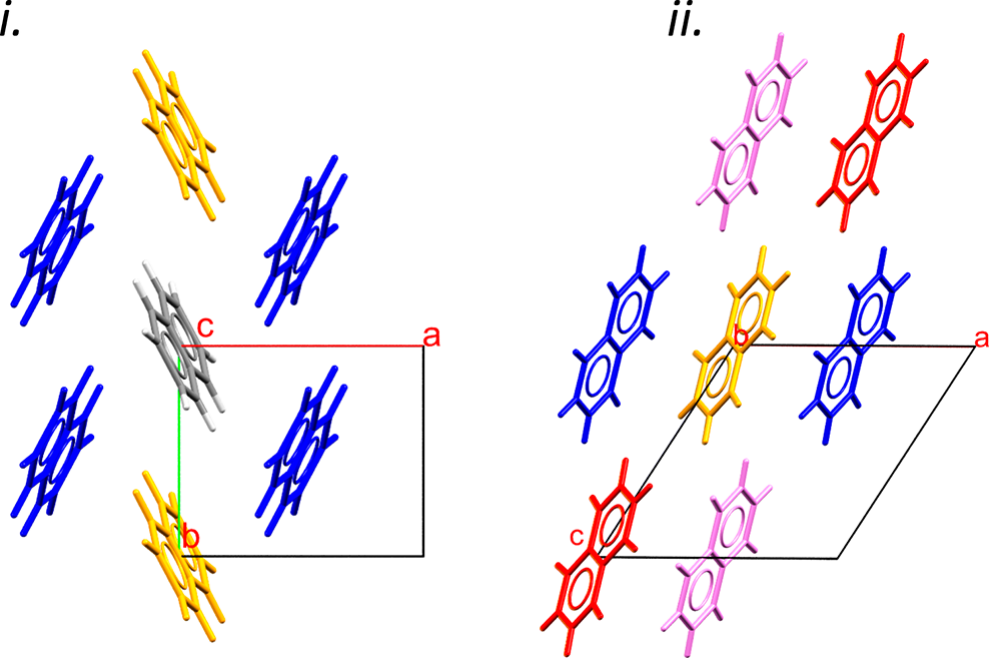
The crystal structure of naphthalene. (i) Viewed down the *c*-axis. (ii) Viewed down the *b*-axis. Molecules are color coded to match pixel contact graphs below with respect to the central gray molecule.

The structure of naphthalene at ambient pressure (NAPHTA47) can be viewed as consisting of layers formed in the *ab* planes [Figure 3(i)]. Within the layers, each molecule interacts with six nearest neighbors. Four of these interactions are through a set of equivalent CH−π contacts [Figure 3(i)] [contact #1; shortest H···C distance = 2.84 Å, “herringbone” angle between the planes of the naphthalene molecules = 51.51°, centroid–centroid distance = 5.099 Å, energy −17.2 kJ mol^–1^, according to the pixel method]. Two longer contacts [#2 in Figure 3(i)] form, in which the molecules are related by lattice translations along *b*. In the two longer contacts, the H-atoms of one molecule lie above the C-atoms in the other, but the displacement is too large to describe it as a “stacking interaction,” and it is probably best regarded as a non-specific dispersion interaction.^34^ It is, nevertheless, almost as strong as contact #1 (centroid–centroid = 5.984 Å, energy = −15.6 kJ mol^–1^). The layers stack along *c*, each molecule interacting with three molecules in the layers above and below, featuring close H···H contacts [Figure 3(ii)] [shortest H···H distances = 2.40 Å, two equivalent contacts labelled #3, and one unique contact labelled #4, centroid–centroid = 7.913 and 8.675 Å, energies −7.6 and −6.4 kJ mol^–1^, respectively], to give an overall molecular coordination number of 12. The stacking follows an ABC sequence, and the structure topologically resembles cubic close packing.

**Figure 4:**
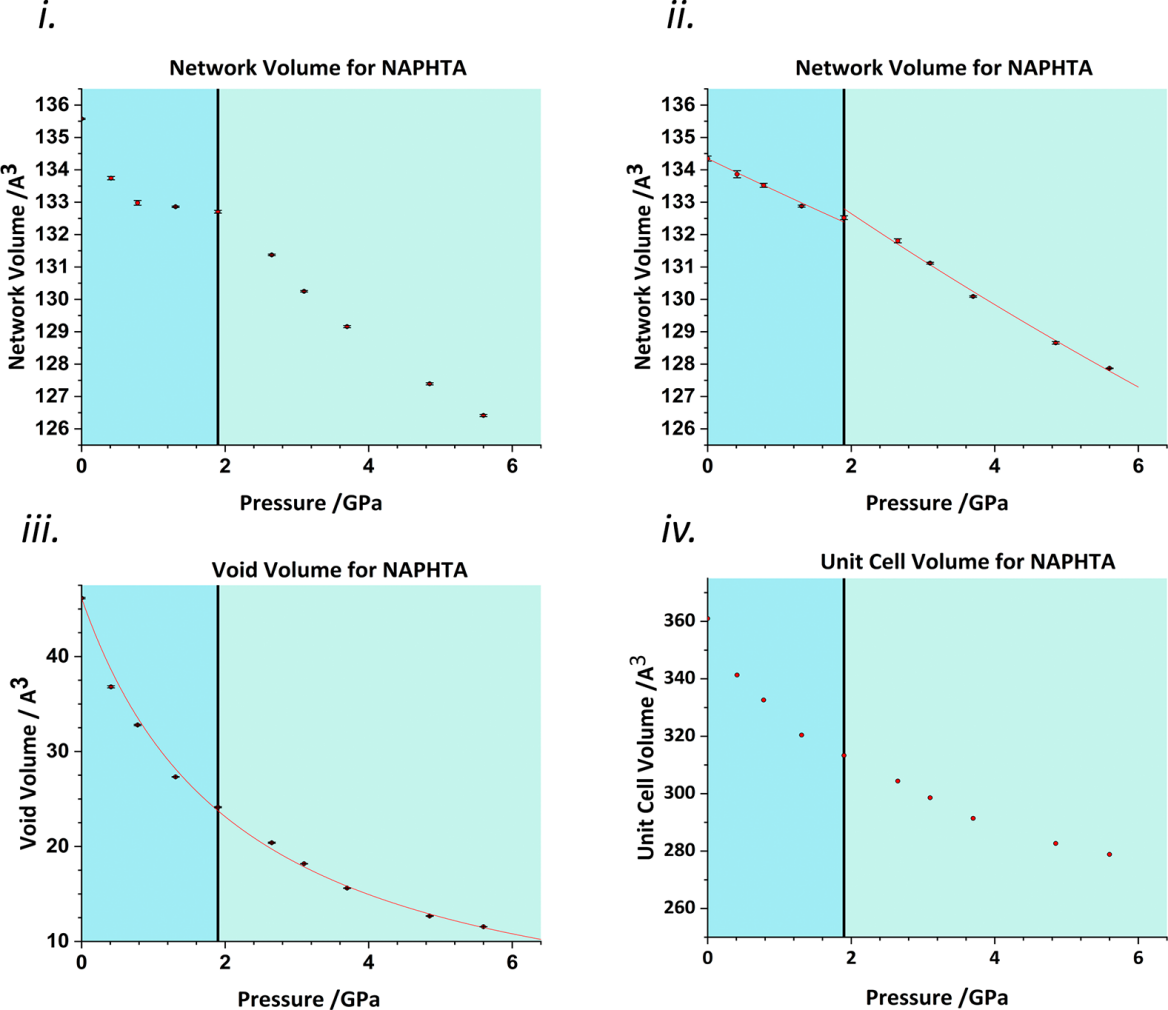
(i) Network volume plot derived using data from the high-pressure powder diffraction study of naphthalene by Likhacheva *et al*.^32^ (ii) Network volume plot derived using DFT-optimized data. Second-order Birch–Murnaghan EoSs have been fitted before and after 1.90 GPa. (iii) Void volume plot using DFT-optimized data. A third-order Vinet EoS has been fitted across the full pressure range. (iv) Unit cell volume as a function of pressure. Black vertical lines mark the discontinuity discussed in the text.

Analysis of effect of pressure on the crystal structure of naphthalene revealed that after the initial compression of the network volume by 2.5 Å^3^ between 0 and 1 GPa, the trend levels off between 1 and 2 GPa, followed by a collapse above 2 GPa (Figure 4(i)). This trend was eventually traced to a parallel decrease in the volume of the naphthalene molecules themselves rather than that in the intermolecular contacts, for example, C–C bond distances span a range of 1.385(4)–1.448(3) Å at ambient pressure and 1.339(6)–1.400(7) at 5.60 GPa. While changes in covalent distances are possible with pressure, in other structures,^23,35^ they tend to be of the order of 0.02 Å at around 7 GPa, and it appears that change in the bond distances and molecular volume are related to instabilities in the Rietveld refinements used in structure analysis. The crystal structures were therefore optimized using periodic DFT. Application of volume analysis to the optimized structures showed a more subtle but nevertheless clear change in the gradient of the network volume [Figure 4(ii)] with the points between 0 and 2 GPa lying on one line, and those between 2.65 and 5.60 GPa lying on another, steeper, line. Fitting of the network volume using a second-order Birch–Murnaghan EoS before and after 1.90 GPa revealed a reduction in the network bulk modulus from 125(4) to 82(4) GPa. The signs of the transition are less obvious in the void and total volumes [Figure 4(iii,iv)]. The void volume can be fairly well fitted with a single third-order Vinet EoS [*K*_0_ = 2.1(1) GPa *K*′ = 0.88], though there is some scatter in the fit. The trends indicate that the phase transition involves the network assuming a greater role in accommodating pressure. The onset of additional modes of compression is comparable to a second-order thermal phase transition such as a glass transition in a polymer, in which the heat capacity increases as new vibrational modes become available on heating.

**Figure 5:**
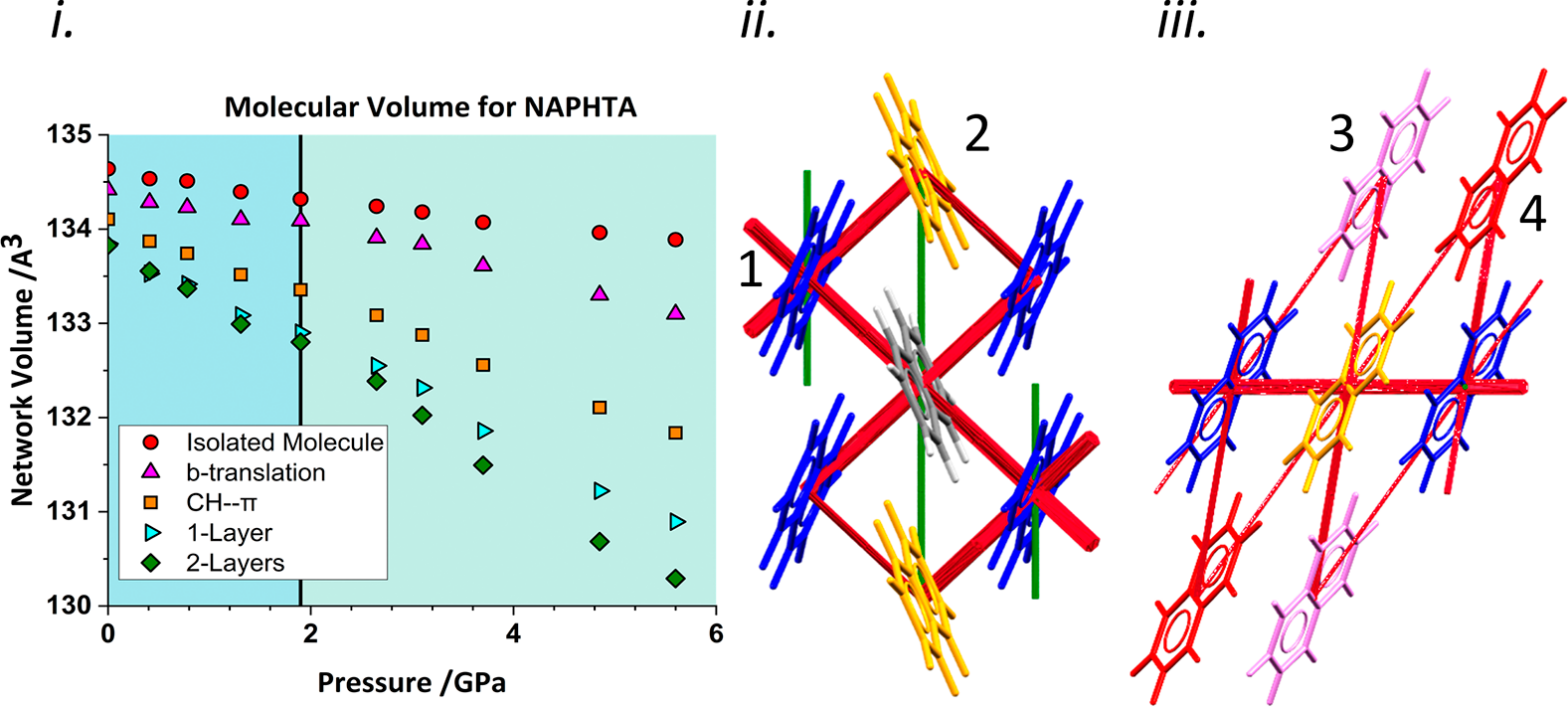
(i) Applications of the CellVol code to molecular subsets in the crystal structure of naphthalene. Data have been scaled to the number of molecules in the subset. For clarity, the isolated molecule is offset by +0.25 Å^3^, the CH−π plot is offset by −0.25 Å^3^, and both the single and double layer are offset by −0.5 Å^3^. Black vertical lines mark the discontinuity. (ii,iii) Energy difference frameworks for the DFT-optimized crystal structures viewed down **c** and **b**, respectively. Struts represent energy differences between ambient pressure and 5.6 GPa. Green and red, respectively, represent stabilization and destabilization on increasing pressure.

It is possible to analyze the origin of the network volume changes by calculating the volume of isolated structural fragments as a function of pressure [Figure 5(i), volumes have been offset vertically for the sake of clarity]. The volume of an individual molecule (calculated as described in ref (23)) decreases linearly by only ∼0.5 Å^3^ up to 5.60 GPa, with the discontinuity seen in the experimental volume no longer present. When the calculation is applied to a fragment of a layer consisting of a central reference molecule and the six molecules connected to it by contacts #1 and #2, a discontinuity in the gradient is observed at the same pressure as in the overall network volume. The volume of the set of five molecules connected by CH−π contacts is approximately linear, while that of the three molecules connected by contact #2 shows a change in gradient above 2 GPa. A calculation applied to a fragment consisting of two layers shows no new features. Since the van der Waals surfaces of the different fragments overlap, the sum of the fragment volumes shown in Figure 5(i) is not equal to the network volume, but the results suggest that the phase transition has its origin in the structure of the layers, particularly in contact #2. This conclusion is in contrast to the original analysis by Likhacheva *et al.*, who suggested that the anisotropic compression was associated with suppression of compression of interlayer C···C contacts.

**Figure 6:**
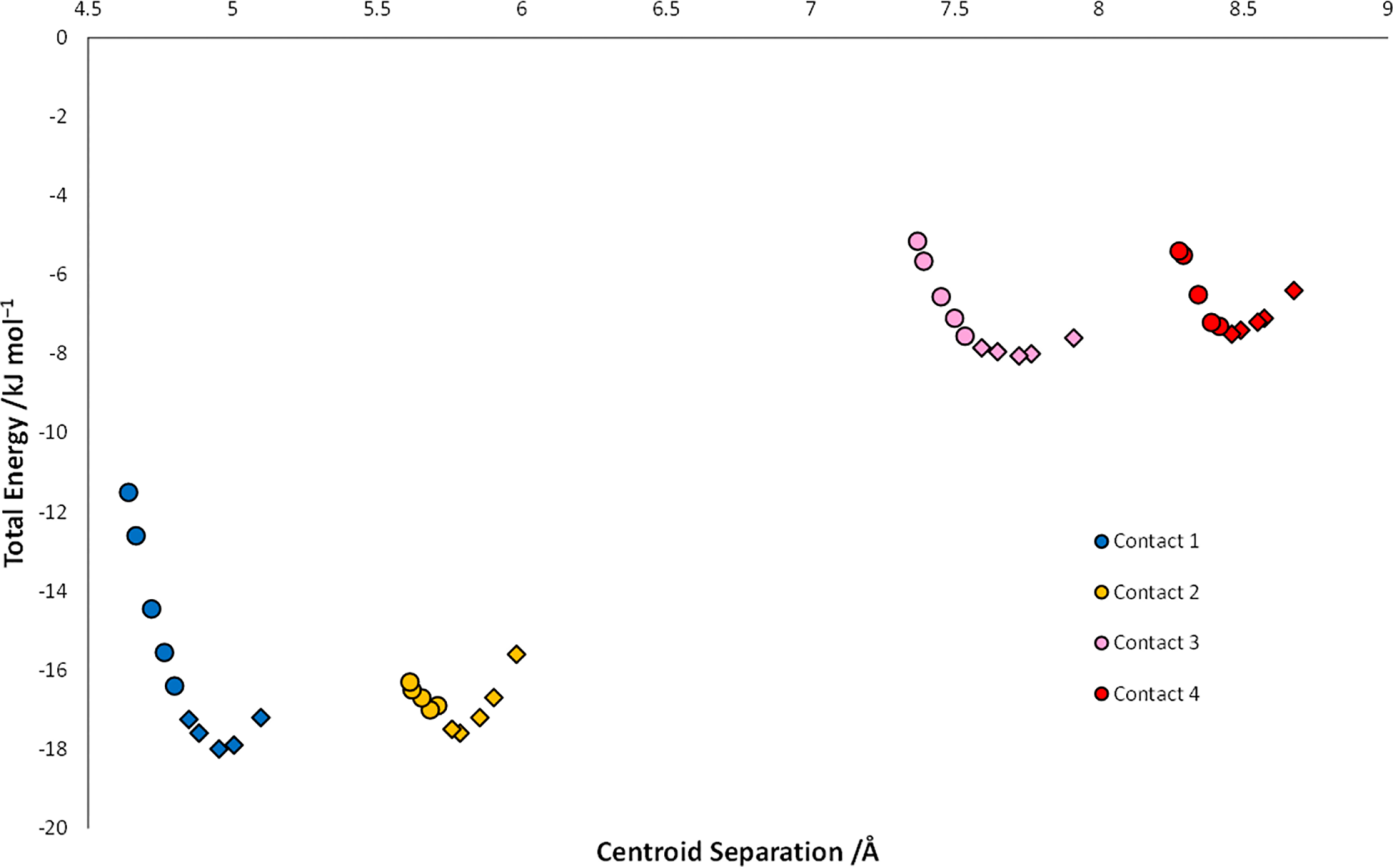
Contact energy *vs* centroid–centroid distance for contacts within the first coordination sphere for the DFT-optimized crystal structure of naphthalene. The colors match those shown in Figure 3. Points before 2 GPa are represented by diamonds, those after are circles.

Contact #2 is a non-specific dispersion contact, and the lack of any characteristic geometrical constraint (such as an optimal herringbone angle in a CH−π contact) makes the interaction very flexible. This feature can be seen in the interaction potentials calculated for the four contacts as a function of pressure using the pixel method (Figure 6). All four contacts are pushed into progressively less stabilizing regions of their potentials between ambient pressure and 5.60 GPa. Destabilization occurs for contacts #1 and #3 first, suggesting that optimization of these directs the crystal structure of naphthalene. The effect is smallest for contact #2, showing that compression of this interaction represents a low energy pathway for accommodating the increased pressure. The change in energy in the contacts is still clearer in an energy difference framework (Figure 5(ii,iii)),^36^ where the struts represent contact energy differences after compression to 5.60 GPa: contact #2 not only has the thinnest strut, it is also the only contact which is more stable at 5.60 GPa than it was at ambient pressure.

**Figure 7:**
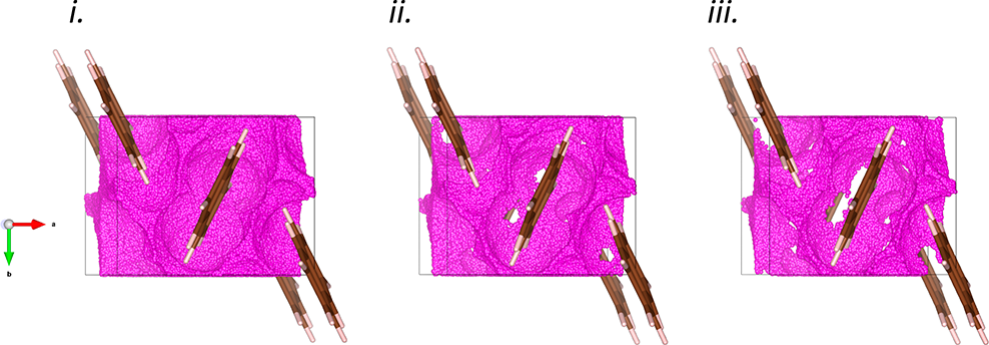
Visualization of void volume calculated with the CellVol code using the VESTA program at (i) 1.31, (ii) 1.9, and (iii) 2.65 GPa. The voids are shown in pink, and white regions correspond to areas where van der Waals surfaces of atoms overlap. Interlayer void volume decreases across the pressure range, disappearing at around 2 GPa.

**Figure 8:**
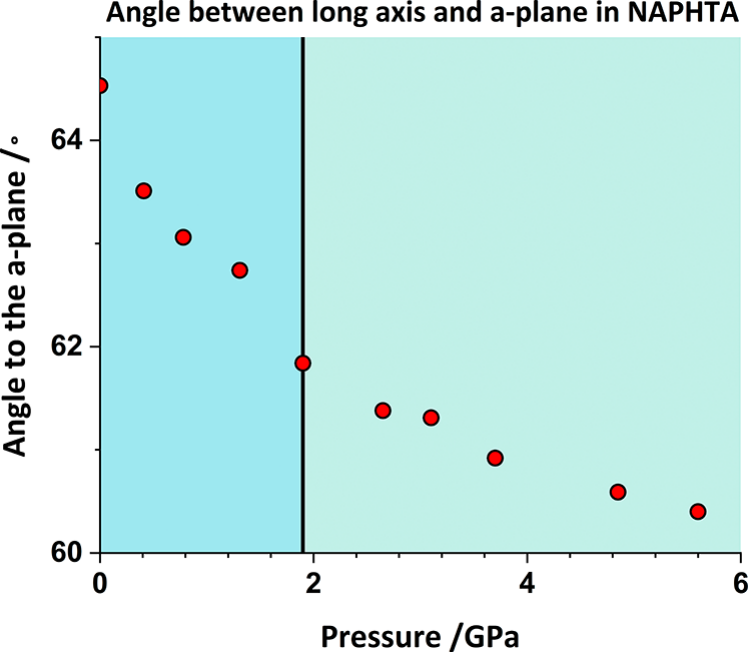
Angle between the long molecular principal axis and the *a* unit cell axis in naphthalene. Values calculated using PLATON. The black vertical line marks the discontinuity.

The points in the curves in Figure 6 are shown as diamonds and circles below and above 2 GPa, respectively, and all four curves show an upward trajectory in energy after the transition, which correlates with the loss of void space as atoms are pushed within the sums of the van der Waals radii (Figure 7^37^). The upward trend appears at slightly lower pressure in the case of contact #1, reflecting the hardening of the herringbone angle identified by Likhacheva *et al.* and the step in the trend of the angle made between the long axis of the molecule and the *a* axis immediately prior to the transition (Figure 8). The destabilization in contact #1 is the result of a marked increase in Pauli repulsion, which increases from 12.5 to 68.4 kJ mol^–1^ over the pressure range. It seems that the transition occurs as the result of the hardening of contact #1, which signals the need to change the mechanism of compression.

### 3.3 Premonitory Behavior

**Figure 9:**
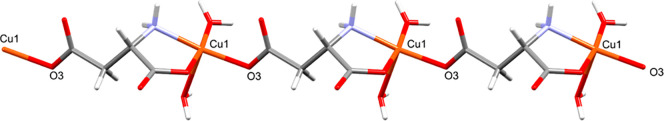
One-dimensional coordination polymer structure of Cu(l-Asp)(H_2_O)_2_] (Asp = aspartate, LEFJAH). Blue: nitrogen, gray: carbon, white: hydrogen, red: oxygen, and orange: copper.

In [Cu(l-Asp)(H_2_O)_2_] (LEFJAH, Figure 9, Asp = aspartate) aspartate ligands bridge Cu(+2) ions, binding at one end in bidentate fashion through the amine and one oxygen of the α-carboxylate group and at the other end through one oxygen atom of the β-carboxylate group to form a one-dimensional coordination polymer.^38^ The square-based pyramidal geometry of the Cu centers is completed by two water ligands, one of which forms in the axial direction of the pyramid. The Cu–N and Cu–O bond distances in the equatorial plane are all below 2 Å, but the distance to the axial water at ambient pressure (LEFJAH01) is 2.311(2) Å. In addition, the second oxygen atom of the β-carboxylate forms a distant interaction [2.925(2) Å] with the Cu, making an angle of 143.16(7)° with the axial water. The Cu···O distances along the axial direction begin to compress above 0.3 GPa, the longer reaching 2.883(6) Å at 0.9 GPa. This was interpreted in terms of a conversion of a long interaction into a primary coordination bond, changing the Cu coordination from [4 + 1] to [4 + 2].^38^ At 6.8 GPa, the shorter axial distance is 2.234(10) Å and the longer is 2.662(13) Å. On increasing the pressure to 7.9 GPa, a phase transition occurs, in which the two distances shorten discontinuously to 2.02(2) and 2.57(2) Å, respectively.

**Figure 10:**
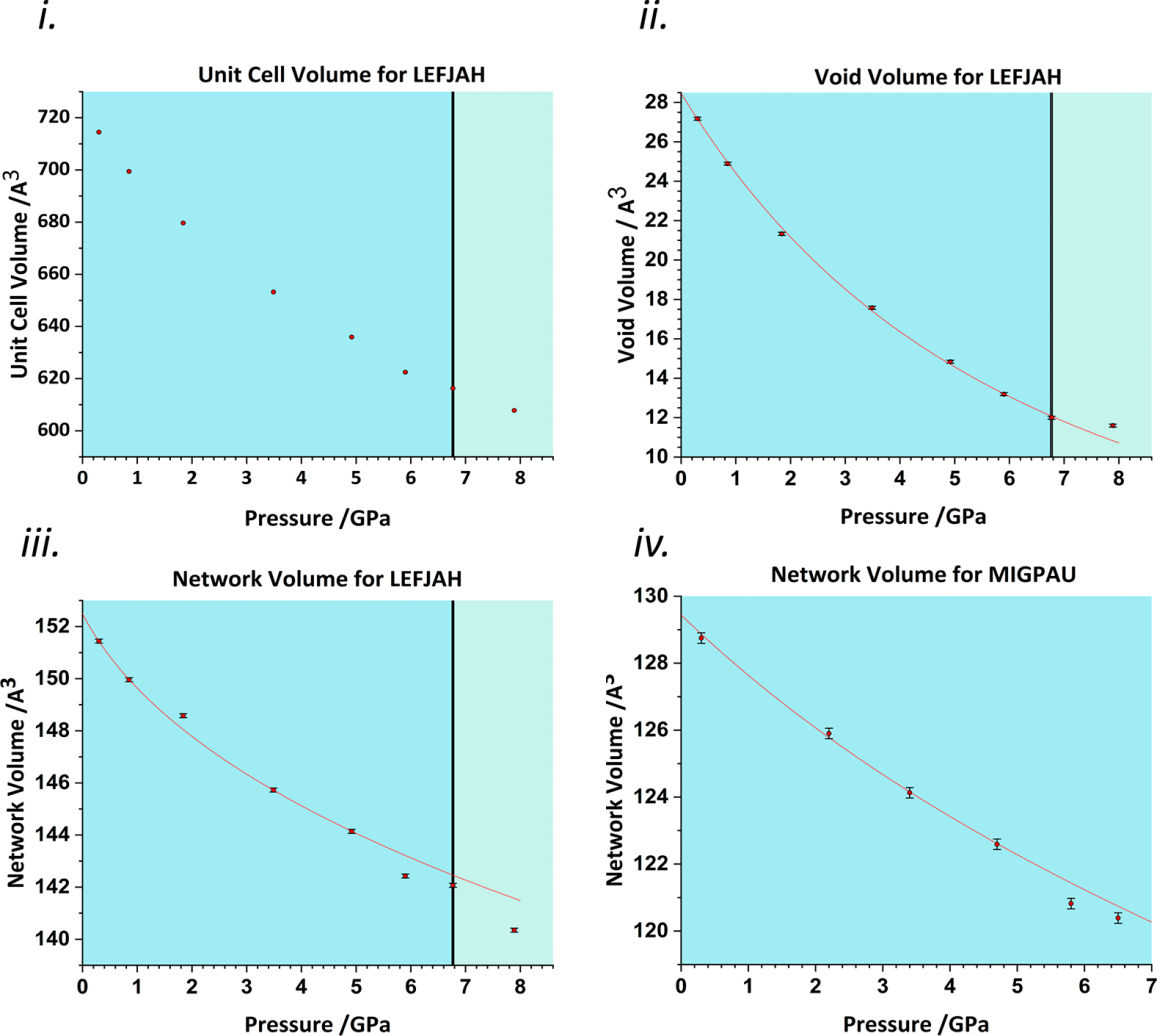
(i) Unit cell volume of LEFJAH against pressure. (ii) Void volume of LEFJAH against pressure with a third-order Vinet EoS fitted to all but the last point. (iii) Network volume of LEFJAH against pressure with a third-order Birch–Murnaghan EoS fitted to the first five points. (iv) Network volume against pressure in MIGPAU with a third-order Vinet EoS fitted to the first four points. This provided a slightly improved statistical fit over second- or third-order Birch–Murnaghan EoS, the more common choice for network plots. Black vertical lines mark the phase transition.

The phase transition produces discontinuities in the unit cell dimensions but not very obviously in the unit cell volume (Figure 10(i)) as an increase in the *a* and *b* dimensions is compensated for by a compression of *c*. The transition is clearly visible in the network and void plots (Figure 10(ii,iii)). The point at 7.9 GPa in the void volume is at *higher* volume than that expected on the basis of a Vinet EoS applied to the points between 0 and 6.8 GPa. At the same time, the network volume decreases by 1.7 Å^3^ per molecule. The transition can therefore be understood in terms of more compact bonding in the network. The bulk moduli of the network and voids between 0 and 6.80 GPa are 38(11) and 6.7(4) GPa, respectively, and the abrupt halt in the reduction in void volume at the transition implies that as in the case of naphthalene, there is a point at which the mechanism of compression switches from the voids to the network. In addition to stepwise compression in the axial Cu–O bonds, Gould *et al.*(38) identified shortening of OH···O H bonds, which connect the polymer chains and a conformational change in the chelate ring which allows compression along the polymer chain as mechanisms through which the network compresses after the phase transition.

The network volume calculations also reveal some structural instabilities before the phase transition, suggesting premonitory structural effects in the bonding. This is shown in Figure 10(iii), in which a third-order Birch–Murnaghan EoS is seen to fit the points between 0 and 4.92 GPa but not those at 5.90 and 6.77 GPa, which fall below the line. In other structures, for example, 3-fluorosalicylaldoxime (MIGPAU), similar features in the network volume (Figure 10(iv)) occur at the end of the pressure series before a destructive phase transition or loss of long-range order which prevented the collection of further diffraction data.^39^ A recent paper on the study of 4-methylpyridine pentachlorophenol (GADGUN) associated a similar feature with a destructive transition, which occurred on further increase in pressure.^40^ Detection of premonitory behavior will aid the interpretation of why transitions take place in some compounds but not in others and for rationalizing the limit of compression while maintaining long-range order.

## 4 General Trends in Packing Coefficients and Bulk Moduli

### 4.1 Variation of Packing Coefficients with Pressure

The packing coefficient *x* of a crystal structure measures the fraction of the unit cell, which is occupied by atoms, and is readily obtained from the network volume
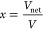
5

**Figure 11:**
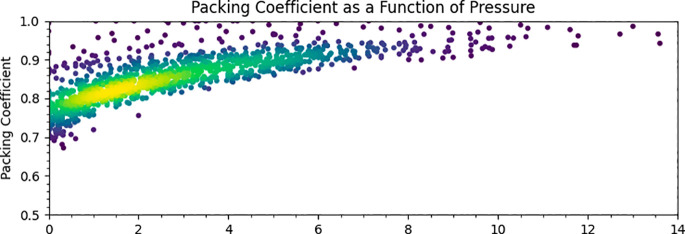
Packing coefficients as a function of pressure for molecular crystal structures.

The packing coefficients of all 1472 structures identified in this study are plotted as a function of pressure and are shown in Figure 11. At ambient pressure, the distribution of *x* mostly falls between 0.6 and 0.8, which is typical for molecular crystals at ambient pressure (see Section 1).^4^ The values increase with pressure, tending toward 1 at above 10 GPa as void space is compressed. This is expected, but the implied total loss of void space reflects the use of constant values of van der Waals radii determined from ambient pressure crystal structures and may obscure a high degree of overlap between molecular van der Waals surfaces in some regions of a structure but less in others. The narrowing with increasing pressure is also a consequence of the importance of the pressure–volume contribution to the free energy, which places a premium on efficient packing as pressure increases.

### 4.2 Contributions of Networks and Voids to Overall Compressibility

The bulk modulus (*K*, eq 3) is a measure of the compressibility or hardness of a material. It has units of pressure, and some typical values for H-bonded molecular solids are 14.0(5) and 11.6(6) GPa for l-histidine I and II, respectively,^23^ and 13.1(6) GPa for l-alanine.^41^ Values for van der Waals solids usually fall below 10 GPa, for example, benzene, bianthrone, and Ru_3_(CO)_12_ with bulk moduli 5.5(7), 8.1(5), and 6.6 GPa, respectively.^42−44^ Ionic salts such as NaCl and CaF_2_ have values of 25 and 82.0(7) GPa,^42,45^ and those for moderately hard metals such as scandium and titanium are 57 and 110 GPa, respectively.^46^ The bulk modulus of diamond is 445 GPa.^47^ The pressure derivative, *K*′, is dimensionless and has typical values between 4 and 10 for molecular solids.

The examples discussed above show that the magnitudes of compression exhibited by voids and networks are very different. The contributions made by the voids and network to the compression of the overall volume can be analyzed by recognizing that since the total volume *V* = *V*_net_ + *V*_void_, where *V*_net_ and *V*_void_ are the network and void volumes, then
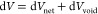
6

Substituting eq 6 into the inverted form of eq 3 yields eq 7.
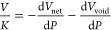
7

Multiplication of the first and second terms on the right-hand side, respectively, by *V*_net_/*V*_net_ and *V*_void_/*V*_void_ gives eq 8
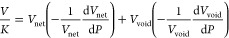
8

By analogy with eq 3, the first term in brackets on the right-hand side of eq 8 can be described as the reciprocal of the network bulk modulus *K*_net_, and the second, the reciprocal of the void bulk modulus *K*_void_, so that the overall bulk modulus (*K*) is related to *K*_net_ and *K*_void_ by
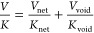
9

Although the concept of the “bulk modulus of a void” seems unphysical, it exists within a model in which the total volume of a unit cell is described in terms of occupied and unoccupied space. It arises as a term in eq 8, which expresses the different responses of these spaces to pressure. Whereas the overall bulk modulus is a precisely defined quantity expressed in terms of pressure and volume (eq 3), its partitioning into networks and voids is a matter of definition (here, using van der Waals radii), and so *K*_net_ and *K*_void_ do not have the same formal thermodynamic status as *K*. Nevertheless, values can be calculated by fitting *V*_net_ and *V*_void_ to an EoS and are useful for comparative purposes.

Some representative values of *K*_net_ and *K*_void_ are given in Table 1, and a more extensive listing for 129 different compounds, including values of the ambient pressure volumes, bulk moduli, and their pressure derivatives, is available in Supporting Information (Table S1). A discussion of the fitting and the quality of the fits can be found in Section S2 of Supporting Information. After some experimentation, we found that network curves were generally fitted well with a second- or third-order Birch–Murnaghan EoS. The volumes of the much softer voids were usually better modeled with Vinet EoSs, as is found generally for the total volumes of soft solids and materials under very high compression.^48^ For some structures, it proved very difficult to fit *V*_net_, and in these cases, *K*_net_ was estimated using eq 4.

**Table 1:** Bulk Moduli and Packing Coefficients for Selected Compounds^a^

compound	network bulk modulus/GPa	void bulk modulus/GPa	overall bulk modulus/GPa	packing coefficient
DLSERN-d,l-serine	134(4)	4.9(5)	19(2)	0.7832
IZOXOL-bromo-substituted bisdiselenazolyl radical (R_1_ = ethane, R_2_ = Br)	130(3)*	4.5(1)*	15.0(4)*	0.7828
LALNIN-l-alanine	121(2)*	4.1(1)*	13.1(6)^41^	0.7585
LHISTD-l-histidine orthorhombic	121(5)	3.2(2)	14.0(5)^23^	0.7617
CYSTAC-l-cysteic acid monohydrate	102(8)	5(1)	17(5)	0.7817
LHISTD-l-histidine monoclinic	101(9)	2.9(2)	11.6(6)^23^	0.7561
DANTEN-bianthrone	95(9)	3.5(7)	8.1(5)^44^	0.8195
QAXMEH-ROY OP	87^†^	2.2(1)	4.3(3)^49^	0.7496
QAXMEH-ROY Y	84(4)	2.2(2)	6.0(7)^50^	0.7527
ROMTUJ-bis(3-methoxysalicylaldoximato)-nickel(ii)	80(4)*	2.5(1)*	9.7(10)^51^	0.7961
HUCQED-Ag_2_Cu_2_L_4_ (l = 2-diphenylphosphino-3-methylindole)	67(2)	2.2(5)	6(1)^52^	0.7362
FAYCEP-bis(3-fluorosalicylaldoximato)-nickel(ii)	48(8)	0.9(6)	9.1(17)^51^	0.7836
JEDJAE-Na_5_[Mn(l-tart)_2_]·12H_2_O (1, l-tart = l-tartrate)	45(4)	5.0(6)	23.9(6)^53^	0.8898
VUZLOT-iron trifluoride	40.7(8)*	2.3(1)*	14(1)^54^	0.9153
NIBSOG-KCp (Cp = cyclopentadienyl)	8(2)	1.9(3)	4.9(3)^55^	0.8729

a Full fitting parameters available in Table S1 of Supporting Information. * = *V*_0_ was not refined. ^†^ = Values obtain using eq 4. Packing coefficients were calculated for the lowest pressure structure available from the study. For NIBSOG, the network volume was better fitted to a Vinet EoS rather than a Birch-Murnaghan EoS.

The effect of error propagation in eq 9 can be exemplified by taking typical values and errors of the bulk modulus of the network and void of 100(5) and 3.2(4) GPa, respectively, with an average cell volume of 1000 Å^3^ with a typical packing coefficient of 0.75. The error in the overall calculated bulk modulus [11.7(13) GPa] is 11.1% of the total value. This is very close to the error in the void bulk modulus (12.5%), which provides the most significant contribution to the overall error.

The void bulk moduli fall into narrow range, typically 2–5 GPa. In contrast, network bulk moduli generally lie within the range 40–150 GPa, varying with the class of intermolecular interaction (see below), and are comparable to moderately hard metals.^46^ Although the compressibility of the voids is determined by the distortion of the surrounding network, there appears to be no correlation between void and network bulk moduli (Figure S3 in Supporting Information). The response of a given crystal structure to pressure depends more on the specific relationship between the network and void space than the strength of interactions that are present. However, the narrow range of *K*_void_ implies that no matter how compressible a network is, the way in which the void space adapts to elevated pressure to minimize volume is consistent across a very broad range of molecular solids.

**Figure 12:**
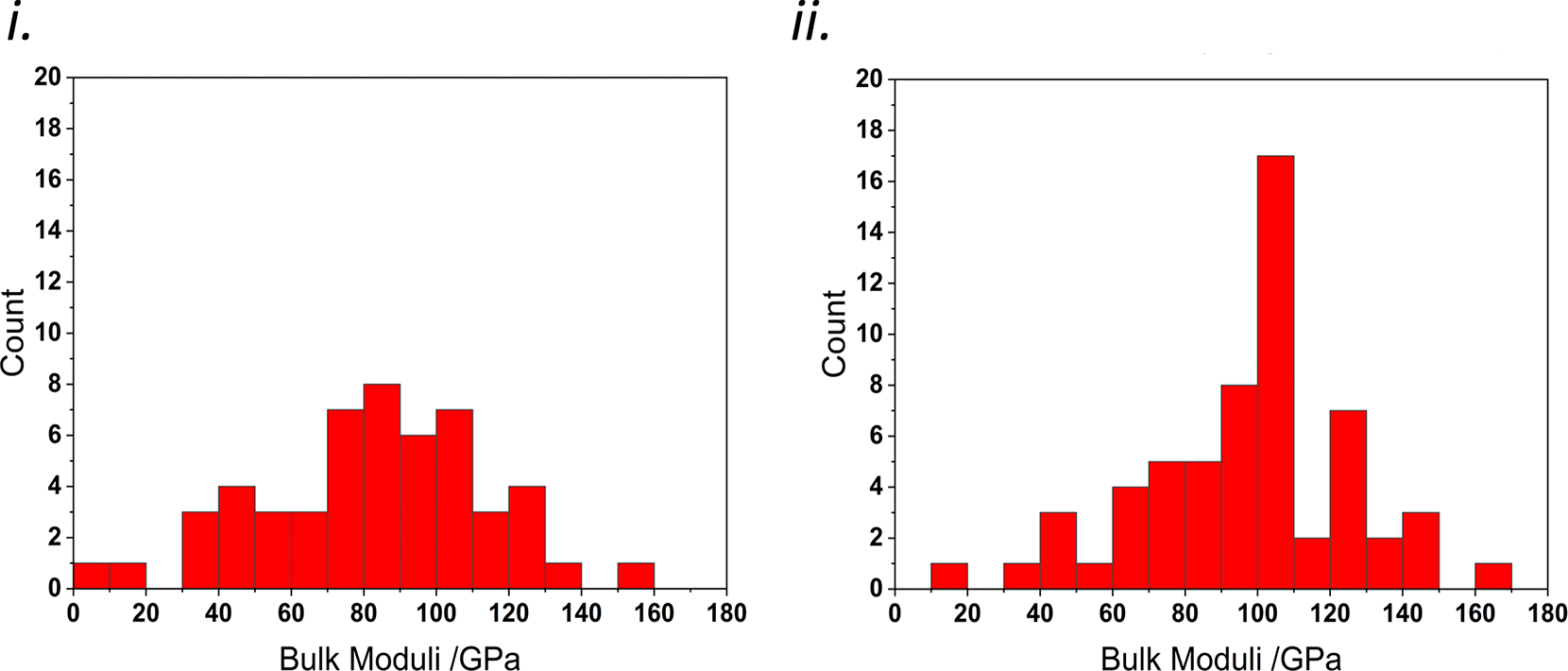
Network bulk moduli histograms for (i) non-hydrogen-bonded compounds and (ii) hydrogen-bonded compounds. Bulk moduli values calculated using eq 4 have been excluded from the plots.

Among molecular materials, H-bonded materials tend to have higher network bulk moduli, for example, l-histidine 121(5) and 101(9) GPa for orthorhombic and monoclinic, respectively, d,l-serine 134(4) GPa, and l-alanine 121(2) GPa, as listed in Table 1. Histograms separating the network bulk moduli of hydrogen-bonded and non-hydrogen-bonded compounds are shown in Figure 12. Hydrogen-bonded compounds produce a tighter distribution centered around 105 GPa, while non-hydrogen-bonded compounds produce a flatter distribution centered around 85 GPa. Hydrogen bonds are the strongest class of intermolecular interaction and are sensitive to geometry, for example, the DH···A angle,^56^ making them less deformable than other classes of intermolecular interactions.

Some non-hydrogen-bonded compounds also have high network bulk moduli. The compound IZOXOL [bromo-substituted bisdiselenazolyl radical (R_1_ = ethane and R_2_ = Br)] has a network bulk modulus of 130(3) GPa. It contains numerous short Se···Se intermolecular interactions and has quite a high packing coefficient (0.783). The typically low bulk modulus of the voids offsets the high network bulk modulus, and the material has an overall bulk modulus of 15.0(4) GPa, which is relatively high for a non H-bonded material.

Van der Waals crystals tend to have lower network bulk moduli; this reflects the lack of a characteristic or sharply defined geometrical signature for dispersion interactions, which can therefore deform with pressure without incurring a high energy penalty, as seen for interaction #2 in naphthalene. An extreme example of this is the compound KCp (Cp = cyclopentadienyl), which features a zigzag polymeric chain with two cyclopentadienyl rings coordinated to each potassium center. Despite the high packing coefficient (0.8729), highly compressible networks and voids, 8(2) and 1.9(3) GPa, respectively, result in low overall bulk moduli (4.9(3) GPa).

Data for the polymorphic compounds l-histidine and ROY are also listed in Table 1. Polymorphic compounds tend to have similar overall bulk moduli, and while there may be some variation in the network bulk moduli and packing coefficients, the void bulk moduli are similar. Orders of the overall bulk moduli of these polymorphs follow packing coefficients, less densely packed polymorphs tending to be the more compressible.

### 4.3 Range of the Bulk Moduli of Molecular Solids

The bulk moduli of minerals, metals, and ceramics span ranges of many 10 s or even 100 s of GPa. Those of molecular materials fall into a much narrower range, typically falling between 5 and 20 GPa. Analysis described above can be used to provide some insights as to why this should be the case.

By substituting eq 5 into eq 9, we obtain
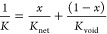
10

Overall bulk moduli for different combinations of the extreme values of *K*_net_ and *K*_void_ in the range *x* = 0.6–0.8 are shown in Table 2. The results reproduce the typical range of bulk moduli for molecular compounds. Since it has a numerically much smaller value than the network bulk modulus, the most significant factor in this calculation is the void bulk modulus. As this lies in a narrow range, so too do the bulk moduli of molecular solids.

**Table 2:** Bulk Moduli Calculated Using Eq 10 from the Range of Network and Void Bulk Moduli Using Typical Packing Coefficients

*K*_net_/GPa	*K*_void_/GPa	range of bulk moduli for *x* = 0.6–0.8
40	2	4.65–8.33
40	5	10.53–16.67
150	2	4.90–9.49
150	5	11.90–22.06

## 5 Conclusions

The methods that we have described enable the changes in overall unit cell volume that occur at high pressure to be decomposed into contributions from the interstitial voids and the network of intra- and intermolecular bonds. The partitioning is based on whether or not random points lie within or outside the van der Waals surfaces of the atoms which compose the structure. In the examples studied, relatively low pressure was seen to be taken up by interstitial voids, but at some point, the network assumed a greater role, and phase transitions could be associated with the onset of an increase in the compressibility of the network.

There is a “compensation” that can occur between the network and void volumes, which means that they are individually more sensitive to phase transitions than the overall volume. Large unit cell volume discontinuities are of course seen in the network and void volumes too, but more subtle effects are also revealed. For example, the literature contains many examples of discontinuities seen in vibrational spectra that seem not to be reproduced by conventional crystallographic analysis but which are seen in the partitioned volumes. This feature was exemplified for naphthalene, where spectroscopic and crystallographic data could be reconciled and unambiguous evidence of a phase transition seen in structural data for the first time since it was discovered in 1938. Detection of effects premonitory to phase transitions and loss of long-range order was also possible.

Network bulk moduli are usually between 40 and 150 GPa, the value reflecting the types of intermolecular interactions present, H-bonded networks being the least compressible. The higher end of these values is comparable to moderately hard metals. Void bulk moduli are over an order of magnitude smaller than network bulk moduli, and surprisingly perhaps, fall into quite a narrow range (usually about 2–5 GPa). Because *K*_net_ ≫ *K*_void_, the value of the overall bulk modulus, which depends on the reciprocals of the component moduli (eq 9), is more strongly influenced by the value of *K*_void_ than that of *K*_net_, and combination of the typical ranges for these quantities with typical packing coefficients recovers the range of bulk moduli seen for molecular solids, which shows much less variation (5–20 GPa) than metals (30–160 GPa), ionic salts (20–90 GPa), or ceramics (50–300 GPa).

The network and void bulk moduli have been shown to be useful parameters for the purposes of comparison of different structures and the same structure in different pressure ranges. The numerical values obtained depend on the method of partitioning, and so, they do not have the same fundamental thermodynamic significance as the overall bulk modulus. It is important to remember that the results of calculations on fragments of networks are not additive because the van der Waals surfaces of atoms and molecules within a network can overlap, so that volume of a network is usually less than the sum of the volumes of the component molecules.

Nevertheless, analysis of the partitioned volume allows the effects of external stimuli to be identified at a “mesoscopic” level between the microscopic level of individual atom–atom distances and the macroscopic level of overall parameters such as the unit cell dimensions. Although the stimulus studied here has been pressure, the approach is equally applicable to crystal structures studied at variable temperatures and with variable compositions, such as in the uptake of guest species by framework materials. The method described should therefore prove extremely useful in the interpretation of crystallographic data collected under varying conditions, including the correlation of effects seen by spectroscopy and other measurements and in characterizing the driving forces of phase transitions.

## Supporting Information Available

Analysis of the number of random points required to reach converged values of the network and void volumes for crystal structures with small and large unit cells; typical computation times as a function of unit cell volume; void and network bulk moduli for molecular materials for which high-pressure structural data available on the CSD; and plot illustrating the lack of any systematic correlation between void and network bulk moduli (PDF)
