# Supplementary material for: Behavior of Occupied and Void Space in Molecular Crystal Structures at High Pressure
Source: Cryst Growth Des. 2022 Mar 22;22(4):2328–41. doi: 10.1021/acs.cgd.1c01427 (PMC9007411; doi:10.1021/acs.cgd.1c01427)
Supplement: Supplementary file 1 — cg1c01427_si_001.pdf [file cg1c01427_si_001.pdf]

Behavior of Occupied and Void Space in Molecular Crystal Structures at High Pressure.

Supplementary Information

Cameron J. G. Wilson,[a] Tomas Cervenka,[a] Peter A. Wood[b] and Simon Parsons*[a]

a Centre for Science at Extreme Conditions, School of Chemistry, The University of Edinburgh, King's Buildings, West Mains Road, Edinburgh, EH9 3FJ, UK

b The Cambridge Crystallographic Data Centre, 12 Union Road, Cambridge, CB2 1EZ UK

Contents

Optimising the Density of Points……………………………………………………………………………………………3

Equations of State Fitting………………………………………………………………………………………………………..4

Comparison of Network and Void Bulk Moduli……………………………………………………………………….27

References……..27

1 Optimising the density of points

In order to optimise the number of random points required for calculations, the variation of the volume was calculated over for different numbers of points using structures with small and large unit cell volumes. Fig. S1 shows extreme examples of the variance for the lowest and highest unit cell volumes likely to be encountered by the program. The graphs featured are for formamide (FORMAM02), one of the smallest-volume molecular solids (unit cell 224 Å^3) and for a porous chromium terephthalate MIL-101 (OCUNAC, unit cell 701860 Å^3) the largest non-disordered structure on the Cambridge Database with coordinates. For both structures network and void values have converged by 1 million points.

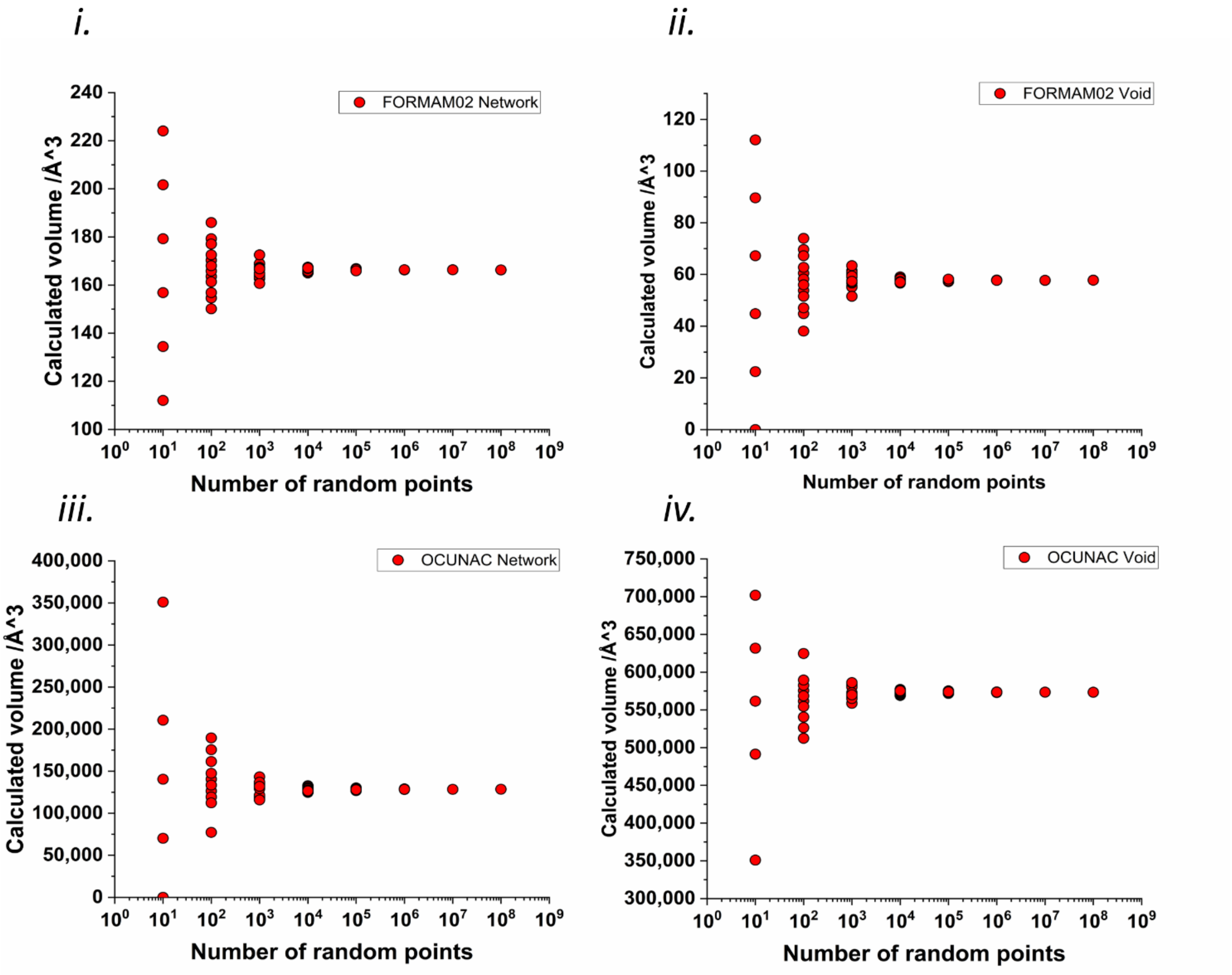

Figure S1: The convergence of the network and void volumes of FORMAM02 and OCUNAC with increasing numbers of random points.

The time taken for calculations using 1 million points per run and achieving a precision of <0.1% for the network volume is shown for a range materials with increasing unit cell volume in Fig S2. The plot is overlaid on a histogram of unit cell volumes of structures in the database. This figure shows that below 1000 Å^3 the calculation time is relatively independent of unit cell

size before becoming linear beyond this. Calculations on ~97% of structures in the Cambridge Database are complete within 5 minutes. The largest non-disordered structure in the database which contains coordinates, OCUNAC, completed in 24 minutes. It is important to note that calculation wall-clock times presented here are a demonstrative guide only and will be dependent on the computer used for the calculations. The values quoted were obtained on a modest desktop PC with an Intel® Core™ i7-9700 CPU with a base speed of 3.00 GHz.

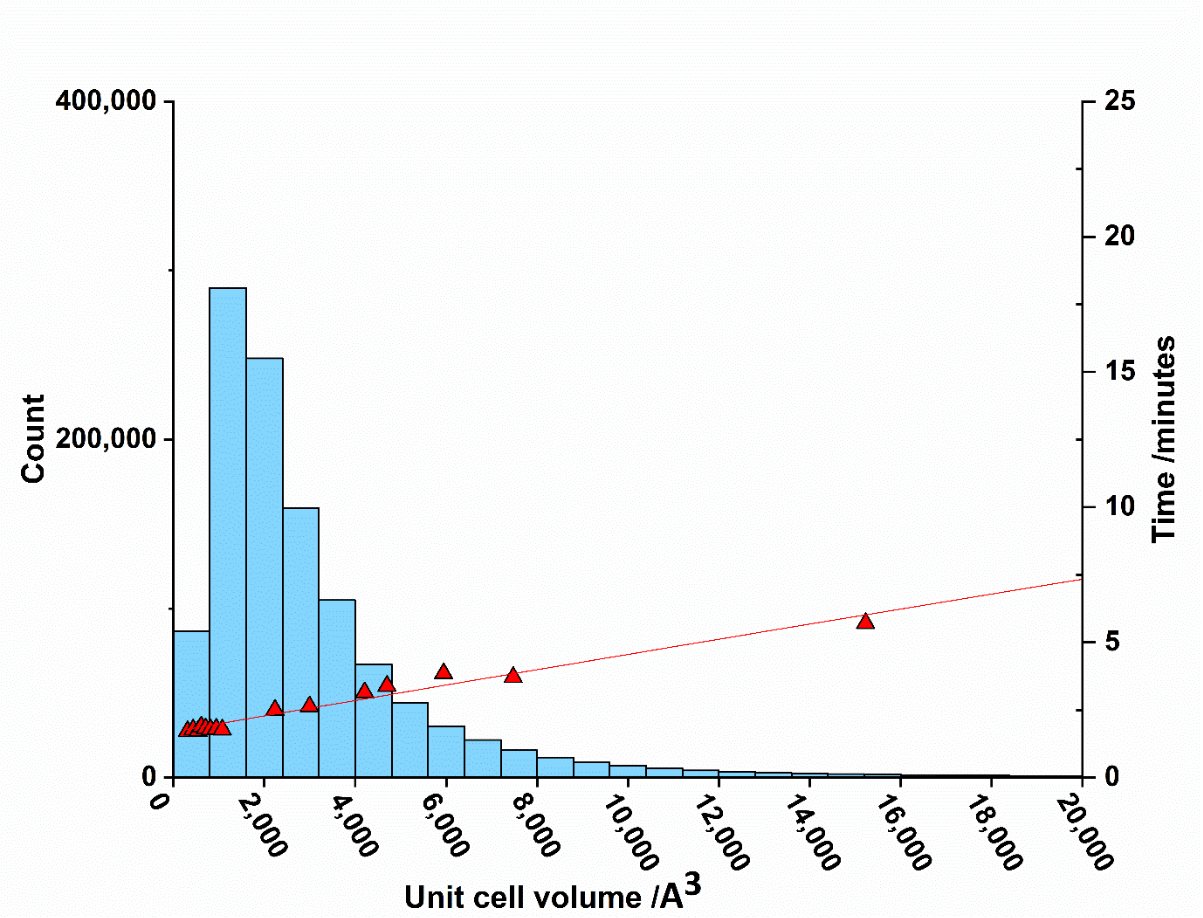

Figure S2: Calculation times for a range of unit cell volumes overlaid on a histogram of unit cell volumes in the CSD. The volume range covers 99.1% of all structures in the CSD.

2 Equation of State Fitting

Equations of state have been fitted to the variation of network and void volume with pressure for all structures on the Cambridge Database which were determined above 0.1 GPa up to 2019. The results are presented in Table S1. Pressures are given in a variety of units in refcode entries on the CSD, but they were all converted to GPa for this study. At least five points in a pressure series were necessary for inclusion, structures which contained no coordinates, had unhandled errors or contained disorder were also removed. The final dataset contained 1472 separate refcodes on 129 different refcode families. Systematic errors were seen when data from several separate studies or even separate diamond anvil cell loadings were combined, and where possible data were taken from contiguous sets of measurements.

The EoSFit program[1] was used to fit volume-pressure data using Birch-Murnaghan or Vinet equations of states (EoSs).[2] Network data were generally fitted well with a 2nd or 3rd order Birch-Murnaghan EoS, whereas void data were modelled better with a (3rd order) Vinet EoS. For some structures network volume was fitted poorly by EoS and for these structures Equation 1 was used,

$$K = -\tilde{V} \times \frac{\Delta P}{\Delta V} \quad (1)$$

where K is the bulk modulus, $\tilde{v}$ is the average volume, ΔP is the change in pressure and ΔV is the change in volume.

Where multiple polymorphs were present for one compound, fitting has been attempted for each of the forms individually. However, for several compounds either a complex structural landscape or incompatible measurement techniques has meant no meaningful fitting was possible. Ambient pressure data points have been used to fix V_0 where possible, and for these entries V_0 is given without a standard deviation. However, for many compounds a significantly improved fit was found by allowing V_0 to vary during fitting and standard deviations are quoted for these entries.

In some cases, a poor EoS fit results from a discontinuous changes of gradient which may imply some form of structural rearrangement such as a change in compression mechanism or phase transition. For the purposes of this list discontinuities (especially in the network curves) that are observed in the absence of an identified first order transition are described as possible second order transitions. Comments have been added in these cases. This behaviour seems to be quite common but difficult to recognise in plots of total unit cell volume versus pressure. The changes in some network volumes for example that of ABUMOZ span only a small range of volume increasing the relative importance of precision. It is important to note the standard deviation used for fitting were taken from the Monte Carlo estimates of network and void volume and do not include any experimental uncertainties and this could account for the generally high values of χ^2. Some fits also yield negative values of K' implying a slightly convex form to the volume-pressure curve.

Table S1: Bulk modulus fitting and calculations for all investigated compounds.

Ref-code	Compound	Volume Type	EoS Type	Order	V_0 /Å^3	K_0 /GPa	K'	χ^2	Comments
ABULIT	o-Vanillin-hydroxylammonium	Network	Birch-Murnaghan	2	149.353	106(2)	4.000	13.03	High χ^2
		Void	Vinet	3	46.988	2.3(2)	0.9(2)	6.36	
ABUMOZ	1,3-Dichlorobenzene	Network	Birch-Murnaghan	2	118.41(9)	110(6)	4.000	4.77	
		Void	Vinet	3	44(2)	2.0(3)	0.7(3)	0.7	
ACRLAC	2-Propenoic acid	Network	Birch-Murnaghan	3	66.9(1)	105(7)	-3(1)	8.96	Fitted above phase transition at 0.87 GPa
		Void	Vinet	3	27(2)	1.9(3)	0.8(1)	2.35	
ACUWOK	cis-Dichloro-(1,4,7-trithiacyclononane-S,S')-platinum(ii)	Network	Birch-Murnaghan	2	223.4(4)	78(3)	4.000	2.52	Data from crystal No. 1 (1.63, 2.88, 3.27, 3.78, 4.19, 5.4 GPa) used for fitting.
		Void	Vinet	3	55(8)	4(2)	0.7(7)	5.23	
ACUWUQ	cis-Dibromo-(1,4,7-trithiacyclononane-S,S')-platinum(ii)	Network	Equation 1		229.587	103.8			
		Void	Equation 1		64.570	7.8			
ACUXAX	cis-Diiodo-(1,4,7-trithiacyclononane-S,S')-platinum(ii)	Network	Equation 1		245.102	100.0			
		Void	Equation 1		63.543	5.5			
ADMANN	α-*D*-Mannopyranose	Network	Birch-Murnaghan	3	144.164	108(7)	16(4)	13.73	High χ^2
		Void	Vinet	3	44.066	4.3(2)	1.1(1)	8.94	
AFUDEL	8-Chloro-4-ethyl-4H-bis((1,2,3)diselenazolo)(4,5-b:5',4'-e)pyridinyl radical	Network	Birch-Murnaghan	2	216.1(4)	103(6)	4.000	6.12	
		Void	Vinet	3	55(3)	5(2)	-0.2(6)	5.47	
ASUQAI	catena-[(μ-iodo)-(6-methylquinoline)-copper(i)]	Network	Birch-Murnaghan	3	196.225	86(2)	-1.7(5)	4.7	Point at 0.28 GPa excluded. Linear network trend.
		Void	Vinet	3	48.074	2.37(6)	0.62(4)	1.62	
BCBANN	syn-1,6:8,13-bis-Carbonyl(14)annulene	Network	Birch-Murnaghan	2	215.076	92(1)	4.000	6.55	

		Void	Vinet	3	68.853	2.2(1)	0.9(1)	4.13	
BEDMIG	4-chloro-N-(propylaminocarbonyl)benzenesulfonamide	Network	Birch-Murnaghan	3	228.9(7)	125(31)	-11(9)	4.02	High esd for K_0
		Void	Vinet	3	73(4)	5(1)	-0.9(5)	2.08	
BENZEN	Benzene	Network	Equation 1		87.163	164.4			Fitted 0.3-1.1 GPa. Relatively few points and high scatter.
		Void	Vinet	3	43(30)	0.4(20)	4(7)	6.58	High esds
BINAPH	cis-1,1'-Binaphthyl	Network							Crystal No.2 - 0.05, 0.1, 0.55, 1.16, 2.21, 2.97 GPa. No meaningful fitting for the network could be completed.
		Void	Vinet	3	77(2)	1.4(2)	1.7(3)	0.8	
BISMEV	(2-Oxo-1-pyrrolidinyl)-acetamide	Network-before							Before phase transition 0.45-0.7 GPa. No meaningful fitting for the network could be completed.
		Void-before	Equation 1		35.657	2.0			
		Network-after	Birch-Murnaghan	2	132.2(2)	99(6)	4.000	5.35	After phase transition 0.45-0.7 GPa. High esd V_0.
		Void-after	Vinet	3	52(25)	0.8(13)	2(2)	8.69	
BOJKAM	catena-(Diaqua-(L-aspartato)-nickel(ii)) monohydrate	Network-before 0.58 GPa	Equation 1		0.000	131.2			Two points only
		Void-before 0.58 GPa	Equation 1		0.000	7.0			
		Network-after 1.26 GPa	Birch-Murnaghan	3	163.500	105(5)	2(2)	2.03	The last point (6.1 GPa) was removed to aid the fitting. V_0 was fixed by linear extrapolation for the network.
		Void-after 1.26 GPa	Vinet	3	42.4(9)	7.5(9)	0.2(3)	1.16	
BOQQUT	3-Aza-bicyclo(3.3.1)nonane-2,4-dione	Network	Birch-Murnaghan	2	141.1(1)	95(2)	4.000	3.36	
		Void	Vinet	3	54(13)	1(1)	1.8(7)	10.50	High χ^2
BZDMAZ	Benzimidazole	Network-α							No meaningful fitting could be made to the polymorph network. 0.06-0.23 GPa
		Void-α	Equation 1		47.395	1.59			
		Network-β	Birch-Murnaghan	2	112.6(2)	78(7)	4.000	3.08	0.62-2.24 GPa
		Void-β	Vinet	3	39(3)	2(1)	2(1)	0.02	

		Network-γ	Equation 1		109.393	69.4			2.23-2.90 GPa
		Void-γ	Equation 1		18.851	4.45			
CABCUD	1-(4-Methylphenylsulfonyl)-3-(hexahydro-1H-azepin-1-yl)-urea	Network-I	Birch-Murnaghan	3	281.6(4)	162(17)	-9(3)	9.01	0.46, 1.33, 2.13, 2.72, 3.00, 4.03, 5.01, 6.10 GPa
		Void-I	Vinet	3	105(7)	1.9(4)	1.5(3)	3.51	
		Network-II	Birch-Murnaghan	3	280.9(7)	145(17)	-4(2)	0.19	3.32, 4.03, 4.72, 5.45, 6,10, 6.83 GPa
		Void-II	Vinet	3	92(18)	3(2)	0.8(6)	0.63	
CARZIF	Methyl 2-(9H-carbazol-9-yl)benzoate	Network-I	Birch-Murnaghan	3	282.241	83(4)	10(2)	1.08	Before phase transition (4.9 Gpa)
		Void-I	Vinet	3	108.087	1.8(1)	2.2(1)	1.02	
		Network-II	Equation 1		268.482	74.7			After phase transition (4.9 GPa)
		Void-II	Equation 1		31.410	6.4			
CEDBUJ	bis(tricarbonyl-(triphenylphosphine)-cobalt)	Network-1							One point only.
		Void-1							
		Network-2	Equation 1		662.626	121.0			Point at 1.3 GPa not fitted due to interpolation in refinement required by poor quality data.
		Void-2	Equation 1		101.477	6.4			
CEGFEA	catena-(bis(μ_2-2-Methylimidazolato-N,N')-di-silver(i))	Network	Birch-Murnaghan	3	235.881	18.8(8)	8.1(7)	1.67	
		Void	Vinet	3	20.351	1.7(1)	-0.2(1)	5.51	
CEKGUU	Trimethylamine	Network-II							Each point is a unique crystal which results in scatter over a small range (1.50-2.40 GPa). No meaningful fitting could be made for the network.
		Void-II	Equation 1		20.739	3.0			
		Network-III	Equation 1		75.421	90.7			Each point is a unique crystal which results in scatter (2.55-3.35 GPa).
		Void-III	Equation 1		14.625	0.82			
CITSOP	2-(2-Fluoropyridin-3-yl)-2-(4-iodophenyl)-2H-3λ^5,2λ^5-[1,3,2]oxazaborolo[5,4,3-ij]quinoline	Network	Birch-Murnaghan	3	325.279	129(9)	-6(3)	5.88	
		Void	Vinet	3	113.236	2.2(1)	1.1(1)	1.15	

COXZAS	6-hydroxy-4,5-dimethyl-2-phenylpyridazin-3(2H)-one	Network							Complex dataset. Multiple phases. No meaningful fitting could be produced.
		Void							
CYSTAC	*L*-cysteic acid monohydrate	Network	Birch-Murnaghan	3	135.72(8)	102(8)	16(4)	0.82	
		Void	Vinet	3	39(2)	5(1)	1.3(6)	16.19	Visibly reasonable fit despite high χ^2.
		Unit-cell	Vinet	3	702(7)	17(5)	10(2)	10.75	
DANTEN	Bianthrone	Network	Birch-Murnaghan	3	353.4(6)	95(9)	1(2)	2.34	
		Void	Vinet	3	105(6)	3.5(7)	0.5(2)	2.94	
		Unit-cell	Vinet	3	941.85	8.1(5)	8.6(5)		Literature value[3]
DEHHEF	(μ_2-η^6,η^6-Benzene)-tetrakis(bis(trimethylsilyl)amide)-di-uranium	Network $P2_1/c$ form	Equation 1		866.377	144.0			Very low number of points
		Void $P2_1/c$ form	Equation 1		326.551	4.4			
		Network *P*-1 form	Equation 1		850.111	160.8			Very low number of points
		Void *P*-1 form	Equation 1		199.518	8.1			
DLALNI	D*L*-Alanine	Network	Birch-Murnaghan	3	81.26(6)	144(10)	-4(2)	4.89	
		Void	Vinet	3	24.6(4)	4.6(4)	1.2(2)	3.14	
DLMAND	D*L*-Mandelic acid	Network-I							Each point is a unique crystal which results in scatter. No meaningful fitting could be produced.
		Void-I	Vinet	3	59(1)	3(1)	-2(3)	0.5	
		Network-II							Each point is a unique crystal which results in scatter. No meaningful fitting could be produced.
		Void-II	Equation 1		38.338	3.2			
DLSERN	D*L*-Serine	Network	Birch-Murnaghan	2	86.88(5)	134(4)	4.000	3.90	
		Void	Vinet	3	24.6(3)	4.9(5)	1.3(3)	2.04	
		Unit-cell	Birch-Murnaghan	3	446(1)	19(2)	9(2)	1.63	

DUCKOB	Decadeutero-n-butane	Network	Birch-Murnaghan	2	82.5(2)	70(4)	4.000	3.67	
		Void	Vinet	3	38(13)	2(1)	0.5(5)	0.16	
EPANIU	3,3'-(3,3,4,4,5,5-hexafluorocyclopent-1-ene-1,2-diyl)bis(2-methyl-1-benzothiophene)	Network-ambient form	Birch-Murnaghan	2	367.8(4)	106(4)	4.000	1.61	0-4.45 GPa
		Void-ambient form	Vinet	3	162(17)	1.9(8)	2.0(6)	1.94	
		Network-HP form	Birch-Murnaghan	2	371(1)	86(5)	4.000	6.85	5.38-8.90 GPa
		Void-HP form	Vinet	3	91(23)	8(5)	-0.3(7)	3.18	
ETDIAM	Ethylenediamine	Network							7 points for 4 polymorphs. No meaningful fitting could be produced.
		Void							
FAPBIH	Dibromo-(1,4,7-trithionane)-palladium(ii)	Network	Equation 1		227.168	116.5			Poor EoS fit
		Void	Equation 1		64.246	9.0			Poor EoS fit
FAWFOY	1,2-bis(2,5-Dimethylthien-3-yl)perfluorocyclopentene	Network-$P2_1/c$ HP form	Equation 1		294.816	132.5			*C*2/c form is disordered and unsuitable for calculation. EoS did not fit well.
		Void- $P2_1/c$ HP form	Equation 1		34.521	8.8			
FAYCEP	bis(3-Fluorosalicylaldoximato)-nickel(ii)	Network	Birch-Murnaghan	2	263.4(6)	48(8)	13(5)	2.08	
		Void	Vinet	3	99(17)	0.9(6)	2.8(8)	4.11	
		Unit-cell	Birch-Murnaghan	3		9.1(17)	10(3)		Literature value[4]
FEGHAA	*L*-Mandelic acid	Network-I	Birch-Murnaghan	3	136.7(1)	104(14)	-34(7)	0.30	Ambient excluded. 0-1.4 GPa
		Void-I	Vinet	3	50(3)	3(1)	1(2)	0.02	
		Network-II							High scatter, no meaningful fitting could be produced.
		Void-II	Equation 1		29.853	4.6			1.55-2.6 GPa
FEROCE	Ferrocene	Network-I	Birch-Murnaghan	2	153.5(2)	108(7)	4.000	6.83	Ambient excluded, 0.88-2.9 GPa
		Void-I	Vinet	3	42(1)	4.2(5)	-0.9(2)	0.27	
		Network-I’	Equation 1		149.328	85.5			3.24-11.6 GPa
		Void-I’	Vinet	3	34(22)	4(3)	-0.3(4)	12.50	

FIKKOC	catena-((µ-iodo)-(µ-methyl 2-aminopyridine-4-carboxylate)-copper)	Network	Birch-Murnaghan	3	188.780	92(3)	-0.08(90)	5.81	
		Void	Vinet	3	50.840	2.7(1)	0.59(8)	3.43	
FOKNEY	Dodecacarbonyl-tri-ruthenium	Network	Birch-Murnaghan	2	328.1(7)	79(3)	4.000	12.44	Ambient excluded
		Void	Vinet	3	104.939	1.8(2)	1.0(2)	19.15	Ambient included, large χ^2.
FOXNUB	Guanidinium tetrafluoroborate	Network- *R*3m form							No meaningful fitting could be produced.
		Void-*R*3m form	Vinet	3	38.490	1.5(5)	2(2)	0.16	
		Network-*R*3	Birch-Murnaghan	2	107.9(2)	123(18)	4.000	3.74	High K_0 esd
		Void-*R*3	Vinet	3	39(16)	1(2)	2(3)	0.85	
FUQLIM	4-chlorophenyl 2-nitrophenyl disulfide	Network	Birch-Murnaghan	2	239.3(2)	98(6)	4.000	5.50	
		Void	Vinet	3	74(2)	2.9(6)	0.1(5)	6.17	
GAFKOO	bis(cyclopentadienyl)-(trimethylphosphine)-(methyl(dichloro)silyl)-(hydrido)-titanium	Network	Birch-Murnaghan	3	323.1(3)	107(8)	-1(2)	3.62	
		Void	Vinet	3	105(4)	2.1(3)	1.1(2)	4.51	
GATLES	cis-Dichloro-(1,4,7-trithiacyclononane-S,S')-palladium(ii)	Network-ambient form	Birch-Murnaghan	2	221.987	75(2)	4.000	11.83	0-4.25 GPa
		Void-ambient form	Vinet	3	60.072	3.8(6)	0.4(5)	15.60	
		Network-Chain polymer	Equation 1		208.969	95.3			4.6-7.68 GPa
		Void-Chain polymer	Equation 1		21.915	8.3			
GEHMAK	4,8-dioxo-4,8-dihydrobenzo[1,2-d:5,4-d']bis[1,2,3]dithiazol-6-ium-3-ide	Network	Birch-Murnaghan	3	172.671	52(4)	5(1)	19.62	
		Void	Murnaghan	3	24.086	2.7(2)	0.24(6)	19.72	Murnaghan EoS used after especially poor fitting of Birch-Murnaghan and Vinet. Compression is in excess of 10%.
GLUCSA	α-D-Glucose	Network-I	Birch-Murnaghan	2	146.9(3)	84(4)	4.000	10.86	0.88-5.33 GPa

		Void-I	Vinet	3	42.6(6)	7.1(5)	-0.3(2)	1.88	
		Network-II							5.54-6.2GPa No meaningful fit could be found for the network
		Void-II	Equation 1		17.554	4.4			
GLUTAM	*L*-α-Glutamine	Network	Birch-Murnaghan	3	128.467	75(13)	26(15)	9.85	
		Void	Vinet	3	30.237	6.1(6)	0.3(4)	7.55	
GLYCIN	Glycine								Not attempted. High number of polymorphs.
GOGJAN	Hexacarbonyl-bis(triphenylarsine)-di-cobalt	Network	Birch-Murnaghan	2	684.681	107(2)	4.000	4.85	
		Void	Vinet	3	311(32)	0.7(3)	2.5(6)	3.14	
GUMMUW	Croconic acid	Network	Birch-Murnaghan	3	99.799	109(8)	-0.9(32)	7.59	
		Void	Vinet	3	23.247	3.3(1)	0.8(1)	1.11	
HEMTID	tris(μ-3,5-diisopropyl-1,2,4-triazolato-N,N')-tri-gold(i)	Network-I							Very high scatter due to phase transitions. Fitting not possible.
		Void-I							
		Network-II	Birch-Murnaghan	2	557(2)	80(10)	4.000	9.09	
		Void-II	Vinet	3	194(13)	3.6(14)	0.3(9)	3.91	
HUCQED	tetrakis(μ_2-2-(diphenylphosphanyl)-3-methyl-1H-indol-1-yl)-di-copper(i)-di-silver(i)	Network	Birch-Murnaghan	2	1323(1)	67(2)	4.000	1.50	
		Void	Vinet	3	554(23)	2.2(5)	2.1(5)	0.28	
		Unit-cell	Birch-Murnaghan	3		6(1)	11(2)		Literature value[5]
IBPRAC	2-(4-Isobutylphenyl)propionic acid	Network	Equation 1		213.970	133.5			Results for network appear to suggest a second order transition. EoS fitting to the entire series was especially poor because of this.
		Void	Vinet	3	92.625	1.74(7)	1.7(1)	0.55	
IFIZIG	(S)-4-Sulfo-L-phenylalanine monohydrate	Network-I	Equation 1		211.520	98.8			Two data points only (0.2, 1 GPa)
		Void-I	Equation 1		74.039	4.8			
		Network-II	Birch-Murnaghan	2	212.2(3)	104(4)	4.000	2.61	2.5-6.9 GPa
		Void-II	Vinet	3	59(5)	4(1)	0.9(4)	0.89	

IMAZOL	Imidazole	Network- α	Birch-Murnaghan	3	66.23(7)	146(21)	-21(7)	3.81	High K_0 esd.
		Void- α	Vinet	3	26(1)	1.3(3)	1.4(3)	0.99	
		Network-β	Birch-Murnaghan	3	66.5(1)	106(25)	-8(8)	9.03	A lot of scatter. High K_0 esd.
		Void-β	Vinet	3	20(1)	2.1(5)	0.8(4)	1.99	
IMEGIR	D*L*-Alaninium semi-oxalate monohydrate	Network-I	Birch-Murnaghan	2	159.399	92(4)	4.000	2.56	0-1.5 GPa
		Void-I	Vinet	3	55.612	3.5(4)	2(1)	0.08	
		Network-II	Birch-Murnaghan	2	160.3(4)	101(7)	4.000	6.54	2.4-5.4 GPa
		Void-II	Equation 1		29.845	6.3			
IPIXUB	bis(4-methylphenyl) disulfide	Network-α	Birch-Murnaghan	2	236.957	154(6)	4.000	1.02	0-1.52 GPa
		Void-α	Vinet	3	81.249	2.9(4)	0.2(7)	0.31	
		Network-β	Birch-Murnaghan	2	237.9(4)	94(7)	4.000	0.97	1.72-2.8 GPa
		Void-β	Vinet	3	69(25)	4(6)	0.2(29)	1.07	
		Network-γ	Equation 1		237.153	78.6			Separate polymorph, not related to α or β through HP.
		Void-γ	Vinet	3	80(6)	1.6(5)	1.0(5)	0.21	
IQOMIM	glycine D*L*-tartaric acid	Network	Birch-Murnaghan	3	174.6(1)	109(7)	0.8(18)	2.35	
		Void	Vinet	3	45(1)	2.8(2)	1.1(1)	1.02	
IZOXOL	8-Bromo-4-ethyl-4H-bis(1,2,3)diselenazolo(4,5-b:5',4'-e)pyridin-3-yl	Network	Birch-Murnaghan	3	217.401	130(3)	-0.8(7)	3.99	
		Void	Vinet	3	60.304	4.5(1)	-0.11(4)	2.35	
		Unit-cell	Birch-Murnaghan	3	1110.820	15.0(4)	6.1(3)	1.82	
JAYDUI	propane	Network	Equation 1		62.373	59.9			Scatter in the network graph seems to be high. EoS fitting to the entire series was especially poor because of this.
		Void	Vinet	3	25(13)	4(4)	-0.1(10)	5.36	

JEDJAE	catena-[octakis(μ-aqua)-bis(μ-L-tartrato)-diaqua-manganese-penta-sodium dihydrate]	Network	Birch-Murnaghan	3	543.2(8)	45(4)	3(2)	0.44	
		Void	Vinet	3	75(1)	5.0(6)	0.7(4)	0.24	
		Unit-cell	Birch-Murnaghan	3		23.9(6)	4.2(5)		Literature value[6]
JEKJOY	catena-(tetrakis(μ_2-Diethyldithiocarbamate)-tetra-gold)	Network-α	Equation 1		327.897	4.5			0-0.05 GPa. High scatter in void.
		Void-α							
		Network-β	Birch-Murnaghan	2	644(1)	68(10)	4.000	1.52	High K_0 esd
		Void-β	Vinet	3	270(9)	4(2)	-0.4(37)	0.05	
JOHGIX	(μ_2-1,4-Di-isocyanobenzene)-bis(pentafluorophenyl)-di-gold	Network	Birch-Murnaghan	3	395.281	44(2)	7(2)	2.12	
		Void	Vinet	3	105.958	1.82(9)	1.5(1)	0.79	
KICCOO	Betaine monohydrate	Network	Birch-Murnaghan	3	135.570	103(5)	3(2)	5.86	
		Void	Vinet	3	42.463	3.0(3)	1.0(2)	10.47	
LALNIN	*L*-Alanine	Network	Birch-Murnaghan	3	81.377	121(2)	0.8(2)	3.14	
		Void	Vinet	3	25.906	4.1(1)	0.58(5)	5.27	
		Unit-cell	Birch-Murnaghan	3	431.1(11)	13.1(6)	7.1(3)		Literature value[7]
LEFJAH	[Cu(L-Asp)$(H_2O)_2$] (Asp = aspartate)	Network	Birch-Murnaghan	3	152.5(3)	38(11)	41(16)	0.03	Phase transition at 6.8 GPa. Two points before the transition excluded.
		Void	Vinet	3	28.2(3)	6.7(4)	0.31(15)	1.85	All points before transition included.
LHISTD	*L*-histidine	Network-Orthorhombic before transition	Birch-Murnaghan	3	135.62(6)	121(5)	-2(2)	1.09	0-4.5 GPa

		Void-Orthorhombic before transition	Vinet	3	43.9(6)	3.2(2)	0.81(14)	1.29	
		Network-Orthorhombic after transition	Birch-Murnaghan	2	137.3(4)	83(5)	4.000	1.94	4.5-6.6 GPa
		Void-Orthorhombic after transition	Vinet	3	25(14)	8(12)	-0.4(23)	3.81	
		Network-Monoclinic before transition	Birch-Murnaghan	3	135.55(6)	101(9)	3(6)	1.05	0-3.1 GPa
		Void-Monoclinic before transition	Vinet	3	44.0(5)	2.9(2)	0.8(2)	0.45	
		Network-Monoclinic after transition	Equation 1		130.942	139			3.1-6.8 GPa
		Void-Monoclinic after transition	Vinet	3	25(3)	9(3)	-0.7(6)	8.39	
LILRIJ	4-oxo-3H,4H-benzo[1,2-d:5,4-d']bis[1,2,3]dithiazol-3-yl radical	Network-α	Equation 1		176.570	31.5			Two points only
		Void-α	Equation 1		41.247	1.7			
		Network-β	Equation 1		153.577	65.0			Two points only
		Void-β	Equation 1		15.346	2.2			
		Network-γ	Equation 1		144.809	94.7			Three points only. Void very close to zero volume.
		Void-γ	Equation 1		2.737	10			
LIQLIH	catena-((μ_2-pyrazine)-diaqua-difluoro-copper(ii))	Network-I							Multiple sources and crystals which results in scatter. Meaningful fitting was not possible.
		Void-I							
		Network-II							
		Void-II							
		Network-ε							One point only. 3.3 GPa
		Void-ε							
LIZFIK	(μ_2-4,4'-Bipyridine)-bis(2,6-bis(1H-pyrazol-3-yl)pyridine)-tetrakis(isothiocyanato)-di-iron(ii) methanol solvate	Network-$P2_1/n$ form	Birch-Murnaghan	3	825.413	73(11)	-19(7)	5.94	Results for network appear to suggest a second order transition. EoS fitting to the entire series was especially poor because of this.

		Void- $P2_1$/n form	Vinet	3	295.475	1.7(3)	1.5(7)	2.23	
LSERIN	*L*-Serine	Network-I	Birch-Murnaghan	2	88.00(3)	121(2)	4.000	0.25	0-4.5 GPa
		Void-I	Vinet	3	24.1(2)	5.0(4)	1.1(2)	0.22	
		Network-II	Birch-Murnaghan	2	89.6(3)	73(4)	4.000	1.14	5.2-7.3 GPa. Fitting EoS to void gave very high esd.
		Void-II	Equation 1		8.658	8.2			
		Network-III							Phase II-III transition was quite unclear. One point only for phase III at 8.1 GPa
		Void-III							
LSERMH	*L*-Serine monohydrate	Network-I	Birch-Murnaghan	2	105.1(2)	107(8)	4.000	5.42	0-4.5 GPa
		Void-I	Vinet	3	30.7(5)	7.6(7)	-0.8(2)	0.00	
		Network-II							One point only. 5.2 GPa
		Void-II							
LTHREO	*L*-Threonine								Not attempted. High number of phase transitions with data collected on multiple sources.
LUSCOR	catena-(bis(μ$_2$-Benzoato-O,O')-(μ$_2$-benzoato-O,O',O')-(dimethylformamide-O)-gadolinium)	Network-I	Birch-Murnaghan	2	433.5(2)	83(2)	4.000	0.11	0-3.73 GPa
		Void-I	Vinet	3	157(5)	1.7(3)	2.1(3)	1.13	
		Network-II							One point only. 5.0 GPa
		Void-II							
MACUFR	catena-[dimethylammonium tris(μ-formato)-copper(ii)]	Network-*I*2/a	Birch-Murnaghan	3	178.505	50(2)	11(1)	1.55	0-5.32 GPa
		Void-*I*2/a	Vinet	3	41.691	5.4(2)	1.4(2)	2.64	
		Network-*P*-1							Disordered data unsuitable for analysis
		Void-*P*-1							

MAGVOG	catena-(tetrakis(μ_2-Ethane-1,2-diamine)-tetra-silver tetranitrate)	Network							Three polymorphs with multiple reloadings data not fitted.
		Void							
MEHNIY	8-Fluoro-4-oxo-3H,4H-benzo[1,2-d:5,4-d']bis[1,2,3]dithiazol-3-yl radical	Network	Birch-Murnaghan	3	175(1)	39(9)	10(4)	10.79	Last point included. Graphs suggest there may be a step down in network volume to this point.
		Void	Vinet	3	28(2)	3.1(4)	-0.2(1)	6.21	
MEWXOE	(6-bromo-2,2'-bipyridine)-(bis(2-(diphenylphosphino)phenyl)ether)-copper(i) hexafluorophosphate	Network	Birch-Murnaghan	2	770(1)	112(6)	4.000	10.00	Results for network appear to suggest a second order transition. EoS fitting to the void was especially poor because of this.
		Void	Equation 1		300.116	5.1			
MIGPAU	3-Fluorosalicylaldoxime	Network	Vinet	3	129.3(2)	75(8)	6(3)	1.80	Highest two points excluded from the fit
		Void	Vinet	3	45(3)	1.8(3)	1.1(2)	3.33	Fitted to whole range
MUTKUH	tris(μ_2-Pyrazolato-N,N')-tri-gold(i)	Network	Birch-Murnaghan	3	268.162	38(1)	7.8(6)	1.77	
		Void	Vinet	3	48.293	2.10(8)	0.66(6)	1.63	
NAKCEJ	dichloro-(1,2-phenylenebis(dimethylphosphine))-germanium(ii)	Network	Equation 1		263.638	94.5			Scatter in data did not fit EoS well.
		Void	Equation 1		73.598	5.7			
NAKCIN	dibromo-(1,2-phenylenebis(dimethylphosphine))-germanium(ii)	Network-I	Birch-Murnaghan	2	276.903	57(2)	4.000	4.39	
		Void-I	Vinet	3	102.755	1.4(2)	3.4(6)	1.80	
		Network-II	Equation 1		273.600	65.2			
		Void-II	Equation 1		51.139	7.7			

NALCYS	*N*-Acetyl-*L*-cysteine	Network	Birch-Murnaghan	3	139.3(1)	97(5)	-0.3(13)	1.89	
		Void	Vinet	3	42(1)	2.8(3)	1.0(1)	1.15	
NAOXAL	Sodium oxalate	Network							An ionic compound in which the void volume levels off above the phase transition at 4 GPa. Debatable use of vdW radii. Ionic radii show more conventional behaviour.
		Void							
NAPHTA	Naphthalene	Network-before transition	Birch-Murnaghan	2	134.346	125(4)	4.000	1.56	
		Void- whole range	Vinet	3	46.1450	2.1(1)	0.9(1)	3.65	
		Network- after transition	Birch-Murnaghan	2	135.8(3)	82(4)	4.000	3.05	
NAWYES	dimethylamine	Network-I	Equation 1		57.056	99.5			Three points only
		Void-I	Equation 1		11.412	4.6			
		Network-II	Equation 1		55.335	154.6			Two points only
		Void-II	Equation 1		7.403	3.9			
NEBGUX	1,2-bis(4-Methyl-6-thioxo-1,2,3-thiaselenazolo(4,5-b)pyridin-5-ylideneamino)diselenide	Network-I	Equation 1		359.142	70.8			1.86-3.74 GPa
		Void-I	Equation 1		90.037	3.4			
		Network-II	Equation 1		338.98	83.4			5.09-9.37 GPa
		Void-II	Equation 1		26.978	5.2			
NIBSOG	KCp (Cp = cyclopentadienyl)	Network	Vinet	3	115.8(9)	8(2)	16(2)	3.23	
		Void	Vinet	3	16.3(6)	1.9(3)	1.1(2)	5.45	
		Unit-cell	Vinet	3	1061.6(36)	4.9(3)	11.1(4)		Literature value[8]
NIMRIK	Ethynylbenzene	Network							High scatter.
		Void							
NIRJEE	3-Methylsalicylaldoxime	Network	Birch-Murnaghan	2	143.0(2)	87(3)	4.000	2.26	
		Void	Vinet	3	35(3)	4(1)	0.2(4)	5.10	
NIRJII	3-t-butylsalicylaldoxime	Network-I	Equation 1		191.270	67.1			Two points only

		Void-I	Equation 1		75.406	1.4			
		Network-II	Birch-Murnaghan	2	191.3(3)	96(3)	4.000	4.22	
		Void-II	Vinet	3	64(6)	2.5(8)	1.2(4)	4.21	
NONWES	Dichloro-(1,4,7-oxadithionane)-palladium(ii)	Network-α	Birch-Murnaghan	3	210.7(2)	73(3)	3.4(5)	6.56	Final point excluded.
		Void-α	Vinet	3	46.1(6)	3.4(1)	0.58(4)	1.97	
		Network-β	Birch-Murnaghan	3	210.091	87(2)	1.6(6)	0.92	Phase transition at 6.87 GPa to β' an extensively disordered structure not suitable for analysis here.
		Void-β	Vinet	3	60.044	2.9(1)	0.93(7)	1.44	
		Network-γ	Birch-Murnaghan	3	210.5(2)	83(5)	2.7(9)	6.44	
		Void-γ	Vinet	3	55(2)	2.6(3)	0.7(1)	7.17	
NTROMA	Nitromethane	Network	Equation 1		47.760	82.7			High scatter.
		Void	Vinet	3	27(2)	2(1)	3(1)	1.53	
OHUGUU	bis(μ_2-hydroxo)-bis(μ_2-tetrafluoroborato)-bis(2,2'-bipyridine)-di-copper(ii)	Network	Birch-Murnaghan	2	463.1(3)	71(1)	4.000	1.62	Point at 4.3 GPa excluded
		Void	Vinet	3	119(3)	2.6(3)	1.3(2)	0.38	
OKEZAH	β-D-mannopyranose	Network-I	Birch-Murnaghan	2	144.906	106(4)	4.000	2.22	The point at 2.85 GPa was excluded from fits. It was measured on a different diffractometer.
		Void-I	Vinet	3	49.769	5(2)	1(1)	6.46	
		Network-II	Birch-Murnaghan	2	161(6)	13(4)	4.000	0.94	

		Void-II	Equation 1		27.423	5.0			
OSAVAH	catena-[(μ-hydroxo)-tetrakis(μ-oxo)-boron-calcium-silicon]	Network							An ionic compound in which the void volume levels off at 0 GPa. Debatable use of vdW radii. Ionic radii show more conventional behaviour.
		Void							
PEXNIQ	(2,2'-bipyrimidine)-tetrabromo-rhenium	Network-$P2_12_12_1$	Birch-Murnaghan	2	257.5(1)	101(4)	4.000	0.01	Point at 1.59 GPa excluded
		Void-$P2_12_12_1$	Vinet	3	83(3)	3(1)	2(2)	4.94	
		Network-$P2_1$	Equation 1		251.766	174.4			Two points only.
		Void-$P2_1$	Equation 1		44.784	9.4			
PIWXEY	Cyclohexane-1,2-diamine	Network	Equation 1		127.589	58.5			High scatter.
		Void	Vinet	3	54(7)	2(1)	0.3(11)	5.65	
PRMDIN	Pyrimidine	Network							Three polymorphs with multiple reloadings. Analysis not completed.
		Void							
PYRAZI	Pyrazine	Network-III							Small increase in volume for the network.
		Void-III	Equation 1		21.988	1.3			
		Network-IV	Birch-Murnaghan	2	78.8(2)	99(13)	4.000	2.45	High K_0 esd
		Void-IV	Equation 1		16.796	2.4			
PYRDNO	pyridine N-oxide	Network	Birch-Murnaghan	2	90.5(1)	106(14)	4.000	4.84	High K_0 esd
		Void	Equation 1		19.443	2.6			Fitting not possible.
QAXMEH	ROY	Network-OP	Equation 1		223.268	87.4			
		Void-OP	Vinet	3	78(1)	2.2(1)	0.9(1)	1.86	
		Unit-cell- OP	Birch-Murnaghan	3	1214(4)	4.3(3)	15.3(9)		Literature value[9]
		Network-Y	Birch-Murnaghan	3	224.3(2)	84(4)	-0.5(12)	0.81	
		Void-Y	Vinet	3	68(2)	2.2(2)	0.8(1)	0.99	

		Unit-cell-Y	Birch-Murnaghan	3		6.0(7)	11.1(12)		Literature value[10]
QEYZAY	catena-(bis(μ-glycyl-L-tyrosinato)-zinc)	Network-I	Birch-Murnaghan	2	438(1)	67(10)	4.000	3.56	0-1.52 GPa
		Void-I	Equation 1		94.986	5.9			
		Network-II	Birch-Murnaghan	2	444(4)	48(9)	4.000	7.31	2.48-3.95 GPa
		Void-II	Equation 1		69.381	7.6			
QQQAUG	Chlorothiazide	Network-I	Birch-Murnaghan	2	201.366	109(2)	4.000	5.44	0-4.0 GPa. Void high χ^2.
		Void-I	Vinet	3	63.664	3.9(6)	1.0(6)	13.05	
		Network-II	Birch-Murnaghan	2	200.4(5)	125(8)	4.000	0.01	4.4-5.9 GPa
		Void-II	Equation 1		25.316	6.6			
QQQCIG	Rubrene	Network-I	Birch-Murnaghan	3	521.608	128(3)	-4.9(9)	1.27	0-5.91 GPa
		Void-I	Vinet	3	176.255	2.5(1)	0.9(1)	1.71	
		Network-II							One point only.
		Void-II							
REQCIC	(2,2'-bipyridine)-(9,9-dimethyl-9H-xanthene-4,5-diyl)-bis(di-t-butylphosphane)-copper(i) hexafluorophosphate	Network-ambient form	Birch-Murnaghan	2	758(1)	78(7)	4.000	4.65	
		Void- ambient form	Birch-Murnaghan	2	299(17)	1.2(3)	4.000	3.34	High esd in V_0.
		Network- HP form							One point only
		Void- HP form							

REQCUO	(2,2'-bipyridine)-(9,9-dimethyl-9H-xanthene-4,5-diyl)-bis(di-t-butylphosphane)-silver hexafluorophosphate	Network	Equation 1		763.956	126.9			
		Void	Birch-Murnaghan	2	268(30)	1.0(4)	4.000	10.07	High esd in V_0.
RIJKAZ	catena-[bis(μ-3,3'-(piperazine-1,4-diyl)di(propan-1-amine))-dinitrato-cadmium(ii) dihydrate]	Network							A mixture of phases data not analysed.
		Void							
ROMTUJ	bis(3-Methoxysalicylaldoximato)-nickel(ii)	Network	Birch-Murnaghan	3	307.497	80(4)	2(2)	2.66	
		Void	Vinet	3	78.770	2.5(1)	0.86(9)	0.71	
		Unit-cell				9.7(10)	9.8(16)		Literature value[4]
SALMID	Salicylamide	Network	Birch-Murnaghan	3	121.3(1)	122(8)	-6(2)	0.75	Point for phase II at 0.2 GPa omitted.
		Void	Vinet	3	43(2)	3.7(9)	0.6(5)	8.36	
SALOXM	salicylaldoxime	Network-I	Birch-Murnaghan	2	125.05(9)	89(2)	4.000	0.18	
		Void-I	Vinet	3	38(3)	2.4(6)	0.6(3)	4.06	
		Network-II							One point only.
		Void-II							
SAZZID	bis(4-chloropyridinium) tetrachloro-cobalt(ii)	Network	Birch-Murnaghan	2	314.015	65(1)	4.000	7.31	Possible second order transition.
		Void	Vinet	3	100.113	3.1(1)	0.7(1)	0.75	
SEHHIX	bis(4-chloropyridinium) tetrabromo-cobalt(ii)	Network	Birch-Murnaghan	3	326.0(2)	67(6)	11(4)	1.03	
		Void	Vinet	3	125(4)	1.6(3)	2.6(4)	1.55	
SIMDEA	(dimethylsilanediylbis[4-(3',5'-dimethylphenyl)-7-methoxy-2-methylindenyl])-dimethyl-hafnium(iv)	Network	Birch-Murnaghan	2	657.0(9)	96(6)	4.000	8.24	
		Void	Vinet	3	203(3)	2.7(2)	1.0(2)	0.31	

SOGFOL	bis(Iodo)-(1,4,7-trithionane)-palladium(ii)	Network	Equation 1		241.372	117.4			Results for both network and void appear to suggest a previously unidentified second order transition ca 5 GPa. EoS fitting was especially poor because of this.
		Void	Equation 1		50.674	7.9			
SUCROS	Sucrose	Network-I							High scatter.
		Void-I							
		Network-II							
		Void-II							
THIOUR	Thiourea	Network-V							High scatter.
		Void-V	Equation 1		23.277	1.8			0-0.32 GPa
		Network-VI	Birch-Murnaghan	2	66.2(1)	92(22)	4.000	8.70	High K_0 esd, scatter also high. 0.37-1.75 GPa
		Void-VI	Vinet	3	22(1)	2.6(9)	0.9(11)	0.51	
TUVXAL	4-methylmethcathonone (hydrogen sulfate)	Network-I							Two points only. 0-0.5 GPa. Small increase in volume for the network and void.
		Void-I							
		Network-II	Birch-Murnaghan	2	241.2(3)	106(7)	4.000	2.18	0.88-3.56 GPa
		Void-II	Vinet	3	75(2)	7(2)	1(1)	1.81	
		Network-III							One point only. 4.8 GPa.
		Void-III							
UJIXOC	dichloro-(μ-1,4-phenylenebis(diphenylphosphine))-di-gold	Network	Birch-Murnaghan	3	537.6(5)	62(2)	4.3(5)	3.23	
		Void	Vinet	3	151(3)	2.7(1)	0.64(6)	2.99	
UJUJUF	catena-[tetrakis(μ-cyano)-octakis(pyrazole)-tetracyano-di-manganese-niobium tetrahydrate]	Network	Equation 1		845.467	82.4			Structured network graph. EoS fitting not possible.
		Void	Equation 1		302.392	5.2			

UJUKAM	catena-[tetrakis(μ_2-Cyano)-octakis(1H-pyrazole-N^2)-tetracyano-di-iron(ii)-niobium(iv) tetrahydrate]	Network	Equation 1		837.709	66.4			Gradual spin crossover transition. Equation 1 was applied to the whole range.
		Void	Equation 1		297.501	4.4			
UNIRUF	catena-[bis(μ-dihydrogen-L-tartrato)-tetra-lithium]	Network	Birch-Murnaghan	2	263.2(5)	48(2)	4.000	2.70	
		Void	Equation 1		26.964	5.3			Discontinuity at last point.
UREAXX	Urea	Network-I	Equation 1		52.409	25.4			0.15-0.32 GPa
		Void-I	Equation 1		22.513	5.1			
		Network-III	Birch-Murnaghan	3	52.77(7)	126(18)	-21(6)	1.91	0.55-2.5 GPa
		Void-III	Vinet	3	17(4)	3(5)	2(5)	8.12	
		Network-IV	Equation 1		51.261	44.4			2.75-2.96 GPa
		Void-IV	Equation 1		9.731	0.7			
UTIXAX	Methacrylic acid	Network-I							0-0.39 GPa, quite high scatter but within a small range.
		Void-I	Equation 1		37.401	1.5			
		Network-II	Equation 1		83.587	122.6			0.39-1.2 GPa,
		Void-II	Vinet	3	36(4)	2(1)	0.8(17)	0.36	
		Network-III	Equation 1		82.660	97.3			1.2-3.9 GPa, network calculation from 1.296-3.891 GPa
		Void-III	Vinet	3	29(2)	3(1)	0.3(5)	3.00	
VOFVAN	4-amino-benzophenone	Network-I	Birch-Murnaghan	2	191.850	68(14)	4.000	13.30	0-1 GPa
		Void-I	Equation 1		79.436	1.4			
		Network-II	Birch-Murnaghan	2	190.2(3)	92(4)	4.000	4.89	1-6.2 GPa
		Void-II	Equation 1		24.875	6.2			
VUZLOT	iron trifluoride	Network	Birch-Murnaghan	3	47.611	40.7(8)	7.2(4)	0.09	
		Void	Vinet	3	4.408	2.3(1)	0.31(7)	4.24	

		Unit-cell	Birch-Murnaghan	3		14(1)	12(1)		Literature value[11]
WEMWEQ	2-(trimethylamino)acetic acid	Network	Birch-Murnaghan	3	119.256	117(6)	-5(3)	2.47	
		Void	Vinet	3	34.476	2.5(1)	1.0(1)	0.14	
WEYQAU	catena-[1-ethyl-3-methyl-1H-imidazol-3-ium (m-1,3,5-tricarboxylatobenzene)-manganese]	Network- before 1.7 GPa	Birch-Murnaghan	2	296.7(4)	111(16)	4.000	5.79	Anisotropic compressibility before and after 1.7 GPa were fitted to separate EoS based on comments from the paper.
		Void- before 1.7 GPa	Equation 1		80.869	5.5			
		Network- after 1.7 GPa	Birch-Murnaghan	2	296.7(5)	133(9)	4.000	0.98	
		Void- after 1.7 GPa	Equation 1		56.450	7.5			
WUVSAH	bis(4,5-Trimethylenetetrathiafulvalene-4',5'-dithiolato)-gold	Network	Birch-Murnaghan	3	482(1)	46(4)	7(1)	6.08	
		Void	Vinet	3	91(2)	2.1(1)	0.43(5)	0.59	
XELFEC	perdeuterophosphoric acid	Network- $P2_1/c$							Quite high scatter in the network which restricts fitting but the range of volume is quite narrow. Clear hysteresis in the void plot.
		Void- $P2_1/c$	Vinet	3	22.6(2)	4.3(3)	0.3(2)	0.51	
		Network- $P2_12_12_1$							
		Void- $P2_12_12_1$	Vinet	3	17.6(2)	5.9(4)	0.8(2)	0.71	
YIHHON	2-(Methylamino)acetic acid	Network	Birch-Murnaghan	3	82.121	111(7)	-5(4)	4.09	Data from slow compression analysed. Graphical results suggestive of a second order phase transition at ca 1.5 GPa.
		Void	Vinet	3	33.519	2.3(1)	0.5(1)	1.11	
YOCZOG	catena-((m-1,3-bis(4-pyridyl)propane-N,N')-dichloro-cobalt(ii))	Network							Data from multiple polymorphs in series.
		Void							
YOSRUU	tris(m_2-3,4,5-Trimethylpyrazolato-N,N')-tri-gold(i)	Network	Birch-Murnaghan	3	427(1)	49(8)	9(4)	2.54	

		Void	Vinet	3	102(4)	3.0(4)	0.4(2)	2.14	

3 Comparison of network and void bulk moduli

Figure S3 shows network bulk moduli plotted against void bulk moduli. There is also no clear correlation in these plots, indicating that a high or low network bulk modulus has very little impact on the compressibility of the void. No matter how incompressible a network, the way in which it 'uses' the void space at elevated pressure to minimise volume is consistent across a very broad range of molecular solids.

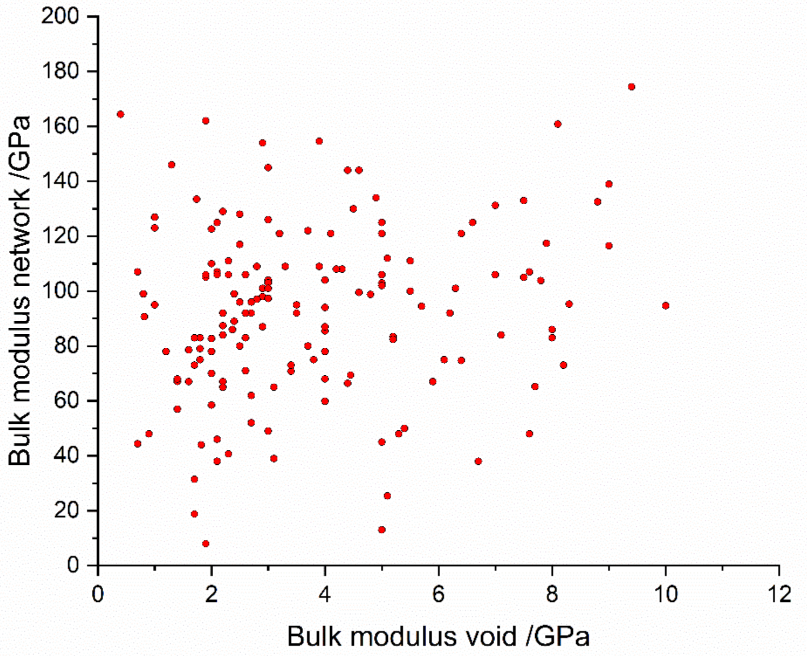

Figure S3: Network bulk moduli vs void bulk moduli for all applicable structures in Table S1. No correlation is seen.

4 References

(1) Gonzalez-Platas, J.; Alvaro, M.; Nestola, F.; Angel, R., EosFit7-GUI: a new graphical user interface for equation of state calculations, analyses and teaching. *J. Appl. Crystallogr.* **2016,** 49, 1377-1382.

(2) Angel, R. J., *High-Temperature and High-Pressure Crystal Chemistry, Mineralogy and Geochemistry, Chap. "Equations of State"*. ed.; Mineralogical Society of America: Washington, DC, 2000; Vol. 41.

(3) Johnstone, R. D. L.; Allan, D.; Lennie, A.; Pidcock, E.; Valiente, R.; Rodríguez, F.; Gonzalez, J.; Warren, J.; Parsons, S., The effect of pressure on the crystal structure of bianthrone. *Acta Crystallogr.* **2011,** B67, 226-237.

(4) Byrne, P. J.; Richardson, P. J.; Chang, J.; Kusmartseva, A. F.; Allan, D. R.; Jones, A. C.; Kamenev, K. V.; Tasker, P. A.; Parsons, S., Piezochromism in Nickel Salicylaldoximato Complexes: Tuning Crystal-Field Splitting with High Pressure. *Chem. Eur. J.* **2012,** 18, 7738-7748.

(5) Jarzembska, K. N.; Kamiński, R.; Dziubek, K. F.; Citroni, M.; Paliwoda, D.; Durka, K.; Fanetti, S.; Bini, R., Impact of high pressure on metallophilic interactions and its consequences for spectroscopic properties of a model tetranuclear silver(I)–copper(I) complex in the solid state. *Inorg. Chem.* **2018,** 57, 8509-8520.
(6) Craig, G. A.; Woodall, C. H.; McKellar, S. C.; Probert, M. R.; Kamenev, K. V.; Moggach, S. A.; Brechin, E. K.; Parsons, S.; Murrie, M., A high-pressure crystallographic and magnetic study of $Na_5[Mn(L\text{-tart})_2]\cdot 12H_2O$ (L-tart = L-tartrate). *Dalton Trans.* **2015,** 44, 18324-18328.
(7) Funnell, N. P.; Dawson, A.; Francis, D.; Lennie, A. R.; Marshall, W. G.; Moggach, S. A.; Warren, J. E.; Parsons, S., The effect of pressure on the crystal structure of L-alanine. *CrystEngComm* **2010,** 12, 2573-2583.
(8) Dinnebier, R. E.; van Smaalen, S.; Olbrich, F.; Carlson, S., Effect of crystal packing on the structures of polymeric metallocenes. *Inorg. Chem.* **2005,** 44, 964-968.
(9) Funnell, N. P.; Bull, C. L.; Ridley, C. J.; Capelli, S., Structural behaviour of OP-ROY at extreme conditions. *CrystEngComm* **2019,** 21, 4473-4483.
(10) Harty, E. L.; Ha, A. R.; Warren, M. R.; Thompson, A. L.; Allan, D. R.; Goodwin, A. L.; Funnell, N. P., Reversible piezochromism in a molecular wine-rack. *Chem. Commun.* **2015,** 51, 10608-10611.
(11) Jørgensen, J.-E.; Smith, R. I., On the compression mechanism of FeF_3. *Acta Crystallogr.* **2006,** B62, 987-992.
